# Predictors of glucocorticoid-free clinical remission in patients with newly diagnosed microscopic polyangiitis and granulomatosis with polyangiitis: a retrospective cohort study using a nationwide registry in Japan (J-CANVAS)

**DOI:** 10.1186/s13075-026-03780-3

**Published:** 2026-03-10

**Authors:** Yusuke Ushio, Risa Wakiya, Hiromi Shimada, Koichi Sugihara, Satoshi Omura, Daiki Nakagomi, Yoshiyuki Abe, Makoto Wada, Naoho Takizawa, Atsushi Nomura, Yuji Kukida, Naoya Kondo, Hirosuke Takagi, Koji Endo, Shintaro Hirata, Naoto Azuma, Tohru Takeuchi, Shoichi Fukui, Kazuro Kamada, Ryo Yanai, Yusuke Matsuo, Yasuhiro Shimojima, Ryo Nishioka, Ryota Okazaki, Tomoaki Takata, Mayuko Moriyama, Ayuko Takatani, Yoshia Miyawaki, Tsuyoshi Shirai, Takafumi Ito, Isao Matsumoto, Toshihiko Takada, Toshiko Ito-Ihara, Takashi Kida, Nobuyuki Yajima, Takashi Kawaguchi, Yutaka Kawahito, Hiroaki Dobashi

**Affiliations:** 1 Division of Hematology, Rheumatology and Respiratory Medicine, Department of Internal Medicine, Faculty of Medicine, Kagawa University, 1750-1 Ikenobe, Miki, Kagawa 761-0793 Japan; 2 Inflammation and Immunology, Graduate School of Medical Science, Kyoto Prefectural University of Medicine, Kyoto, Japan; 3 Department of Rheumatology, University of Yamanashi Hospital, Chuo, Yamanashi Japan; 4 Department of Internal Medicine and Rheumatology, Juntendo University, Tokyo, Japan; 5 Center for Rheumatic Disease, Japanese Red Cross Society Kyoto Daiichi Hospital, Kyoto, Japan; 6 Department of Rheumatology, Chubu Rosai Hospital, Nagoya, Japan; 7 Immuno-Rheumatology Center, St. Luke’s International Hospital, Tokyo, Japan; 8 Department of Rheumatology, Japanese Red Cross Society Kyoto Daini Hospital, Kyoto, Japan; 9 Department of Nephrology, Kyoto Katsura Hospital, Kyoto, Japan; 10 Department of Hematology and Rheumatology, Kagoshima University Hospital, Kagoshima, Japan; 11 Department of General Internal Medicine, Tottori Red Cross Hospital, Tottori, Japan; 12 Department of Clinical Immunology and Rheumatology, Hiroshima University Hospital, Hiroshima, Japan; 13 Department of Diabetes, Endocrinology and Clinical Immunology, Hyogo Medical University School of Medicine, Nishinomiya, Japan; 14 Department of Internal Medicine (IV), Osaka Medical and Pharmaceutical University, Takatsuki, Japan; 15 Department of Immunology and Rheumatology, Division of Advanced Preventive Medical Sciences, Nagasaki University Graduate School of Biomedical Sciences, Nagasaki, Japan; 16 Department of Rheumatology, Endocrinology and Nephrology, Faculty of Medicine and Graduate School of Medicine, Hokkaido University, Sapporo, Japan; 17 Division of Rheumatology, Department of Medicine, Showa Medical University School of Medicine, Tokyo, Japan; 18 Department of Rheumatology, Tokyo Kyosai Hospital, Tokyo, Japan; 19 Department of Medicine (Neurology and Rheumatology), Shinshu University School of Medicine, Matsumoto, Japan; 20 Department of Nephrology and Rheumatology, Graduate School of Medical Science, Kanazawa University, Kanazawa, Japan; 21 Division of Respiratory Medicine and Rheumatology, Department of Multidisciplinary Internal Medicine, Faculty of Medicine, Tottori University, Yonago, Japan; 22 Division of Gastroenterology and Nephrology, Tottori University, Yonago, Japan; 23 Department of Rheumatology, Shimane University Faculty of Medicine, Izumo, Japan; 24 Rheumatic Disease Center, Sasebo Chuo Hospital, Sasebo, Japan; 25 Department of Public Health, Nagasaki University Graduate School of Biomedical Sciences, Nagasaki, Japan; 26 Department of Nephrology, Rheumatology, Endocrinology and Metabolism, Okayama University Graduate School of Medicine, Dentistry and Pharmaceutical Sciences, Okayama, Japan; 27 Department of Rheumatology, Tohoku University Hospital, Sendai, Japan; 28 Division of Nephrology, Department of Internal Medicine, Teikyo University Chiba Medical Center, Ichihara, Japan; 29 Department of Rheumatology, Institute of Medicine, University of Tsukuba, Tsukuba, Japan; 30 Department of General Medicine, Shirakawa Satellite for Teaching and Research (STAR), Fukushima Medical University, Shirakawa, Japan; 31 The Clinical and Translational Research Center, University Hospital, Kyoto Prefectural University of Medicine, Kyoto, Japan; 32 Department of Clinical Assessment, Tokyo University of Pharmacy and Life Sciences, Tokyo, Japan

**Keywords:** Glucocorticoids, Glucocorticoid-free, Microscopic polyangiitis, Granulomatosis with polyangiitis, Rituximab, Avacopan

## Abstract

**Background:**

Glucocorticoids (GC) comprise a cornerstone in the treatment of antineutrophil cytoplasmic antibody (ANCA)-associated vasculitis (AAV); however, prolonged GC exposure leads to substantial toxicity and immune system-related complications. Hence, identifying treatment strategies that enable early GC withdrawal while maintaining disease control is of clinical importance.

**Objectives:**

To investigate the proportion and characteristics of patients achieving GC-free clinical remission (GFCR) 48 weeks after treatment initiation in patients with newly diagnosed microscopic polyangiitis (MPA) or granulomatosis with polyangiitis (GPA) and examine their long-term clinical outcomes (up to week 96).

**Methods:**

We conducted a retrospective cohort study using clinical data from a multicenter, nationwide registry in Japan comprising patients with newly diagnosed MPA or GPA and at least 48 weeks of follow-up. GFCR was defined as a Birmingham Vasculitis Activity Score (BVAS) of 0 with complete GC withdrawal. Univariable and multivariable logistic regression analyses were used to identify independent predictors of GFCR at week 48, and a sensitivity analysis using a matched cohort was performed to validate robustness.

**Results:**

A total of 728 patients were enrolled in the registry, of whom 544 were followed for ≥ 48 weeks; among them, 29 (5.3%) achieved GFCR at week 48. Use of rituximab (RTX) for induction therapy and avacopan within 48 weeks were independently associated with achieving GFCR (multivariable analysis, model 1: RTX: odds ratio [OR]: 3.9, 95% confidence interval [CI]: 1.5–10.0; avacopan: OR: 24.3, 95% CI: 5.8–101.9; both *p* < 0.01). Conversely, methylprednisolone pulse therapy was associated with a lower likelihood of achieving GFCR (multivariable analysis, model 1: OR: 0.08, 95% CI: 0.02–0.44; *p* < 0.01). Patient’s demographic and baseline disease characteristics were not predictive of GFCR. In the long-term (weeks 48 through 96), the rates of death, relapse, and serious infection were similar regardless of GFCR status at week 48.

**Conclusions:**

The use of RTX for induction therapy and avacopan within 48 weeks is independently associated with achieving GFCR at week 48 in patients with MPA and GPA, supporting their potential as key components of GC-sparing strategies in AAV. Prospective studies are needed to confirm these results and optimize treatment algorithms to ensure disease control and minimize GC-related toxicity.

**Supplementary Information:**

The online version contains supplementary material available at 10.1186/s13075-026-03780-3.

## Background

Antineutrophil cytoplasmic antibody (ANCA)-associated vasculitis (AAV) is a group of autoimmune conditions, including microscopic polyangiitis (MPA), granulomatosis with polyangiitis (GPA), and eosinophilic granulomatosis with polyangiitis (EGPA), characterized by the production of ANCAs and the development of systemic small vessel vasculitis [1]. AAV causes severe complications, such as rapidly progressive glomerulonephritis, interstitial lung disease, alveolar hemorrhage, and mononeuritis multiplex, resulting in significant morbidity and mortality [2].

The standard treatment for AAV includes glucocorticoid (GC) therapy combined with immunosuppressive agents, such as rituximab (RTX) and cyclophosphamide (CYC) [3–5]; however, long-term GC use is typically associated with a risk of serious complications, such as diabetes, hypertension, osteoporosis, cataracts, glaucoma, and infection [6–8]. As a result, minimizing GC use is now considered a primary goal in the management of AAV, particularly of MPA and GPA. The Plasma Exchange and Glucocorticoid Dosing in the Treatment of Antineutrophil Cytoplasm Antibody Associated Vasculitis (PEXIVAS) trial [9], conducted mainly in Europe and North America, and the Low-dose Glucocorticoid Vasculitis Induction Study (LoVAS) trial [10], conducted in Japan, demonstrated that the treatment efficacy of the reduced-dose GC regimen (starting at 0.5–1.0 mg/kg/day of prednisolone with a more rapid taper) was comparable to the conventional standard-dose GC regimen (starting at 1.0 mg/kg/day with a slower taper). Furthermore, results of the ADVOCATE trial [11] suggested that avacopan, a selective C5a receptor antagonist, may be used as an alternative to GC in the treatment of MPA and GPA. In the ADVOCATE trial, 65.7% of patients in the avacopan group achieved sustained remission at week 52, defined as remission at both week 26 and week 52 without GC use within the preceding 4 weeks.

Although the clinical evidence supporting the use of reduced-dose GC regimens for MPA and GPA is still developing, an important unknown in this sphere is the proportion of patients who can achieve GC-free clinical remission (GFCR) within 1 year after treatment initiation in real-world clinical settings. Furthermore, the patient characteristics or treatment strategies associated with achieving GFCR have not been fully elucidated. Therefore, using data from a nationwide registry in Japan, the present study aimed to determine the proportion and characteristics of patients with newly diagnosed MPA and GPA who achieved GFCR at 48 weeks after treatment initiation and to identify predictors associated with GFCR.

## Methods

### Study design and patients

This multicenter cohort study retrospectively reviewed patient data from the Japan Collaborative Registry of ANCA-Associated Vasculitis (J-CANVAS), a nationwide registry established by 29 hospitals in Japan. The registry enrolled adult (≥ 20 years) patients who were newly diagnosed with AAV or had a severe relapse between January 2017 and March 2023; patients were classified as having MPA, GPA, or EGPA based on the definitions proposed by the 2012 International Chapel Hill Consensus Conference [1] and the European Medicines Agency algorithm [12]. Each patient was followed up from disease onset to any of the following events: death, loss to follow-up, or March 2024. For the present study, we included all patients who were enrolled in the registry and were newly diagnosed with MPA/GPA; patients with a follow-up period of < 48 weeks or missing data on daily GC dose up to week 48 were excluded.

### Data collection

Clinical data from each hospital were retrospectively collected by referring to their clinical records. Data regarding patients’ baseline characteristics (prior to treatment initiation), including demographics (age at diagnosis, sex, body weight, and height), vasculitis subtype (MPA/GPA), comorbidities (hypertension, diabetes, chronic kidney disease, cardiac disease, and cancer), laboratory data (serum albumin, serum creatinine, blood counts for neutrophils and lymphocytes, hemoglobin, serum C-reactive protein [CRP], serum IgG, ANCA serotype [myeloperoxidase [MPO]-ANCA/proteinase 3 [PR3]-ANCA/seronegative]), organ involvement (Birmingham Vasculitis Activity Score [BVAS] 3.0 [13]), treatment details (GC, methylprednisolone pulse, RTX, CYC, plasma exchange [PLEX], high-dose intravenous immunoglobulin, mycophenolate, methotrexate, azathioprine, or mizoribine) and outcomes (death, major/minor relapses, and serious infections) were recorded. All collected data were integrated using an electronic data capture system, Viedoc (PCG Solutions, Uppsala, Sweden).

### Clinical definitions

Major relapse was defined as BVAS > 0 along with “organ-threatening or life-threatening disease in AAV” [14], while minor relapse was defined as BVAS > 0 and not fulfilling the criteria for major relapse. Serious infection was defined as an “infection requiring hospitalization or resulting in prolonged hospitalization” [15].

### Outcomes

The primary study outcome was the proportion of patients who achieved GFCR at week 48 after treatment initiation. Clinical remission (CR) was defined as BVAS = 0. Accordingly, GFCR was defined as BVAS = 0 with complete withdrawal of GC at the time of assessment. The duration of GC discontinuation before the assessment was not evaluated, because longitudinal GC dose data between predefined follow-up visits was unavailable in our registry. Secondary outcomes included potential predictors, such as patient characteristics and treatment regimens, associated with GFCR at week 48, and clinical outcomes for the period between weeks 48 and 96.

### Statistical analysis

Continuous data were presented as median (interquartile range [IQR]), and categorical variables were expressed as frequency and percentage. Between-group comparisons were conducted using the Wilcoxon rank sum test for continuous variables and Fisher’s exact test for categorical variables. Next, univariable and multivariable logistic regression analyses were performed to identify predictors of GFCR; variables with p-values < 0.05 (model 1) and < 0.20 (model 2) in the univariable analysis were used in the multivariable analysis. Odds ratios (ORs) and corresponding 95% confidence intervals (CIs) were estimated. To further confirm the predictors of GFCR, a sensitivity analysis was performed among patients who achieved GFCR versus those that did not using a matched cohort in a 1:3 ratio based on age (± 5 years), sex, AAV subtype (MPA or GPA), and baseline BVAS (± 5). Additionally, the multivariable logistic regression analysis was repeated in the matched cohort to assess the robustness of the findings. All p-values were two-sided, with *p* < 0.05 considered statistically significant. All analyses were conducted using JMP^Ⓡ^ Pro 14 (SAS Institute, Cary, USA) and R (version 4.5.1; R Foundation for Statistical Computing, Vienna, Austria).

### Ethical considerations

This study was approved by the Ethics Committee of the Faculty of Medicine, Kagawa University (approval number: 2025-067), and was conducted in accordance with the principles of the Declaration of Helsinki. The requirement for written informed consent was waived by the committee because of the retrospective nature of the study.

## Results

### Patient flow

**Fig. 1:**
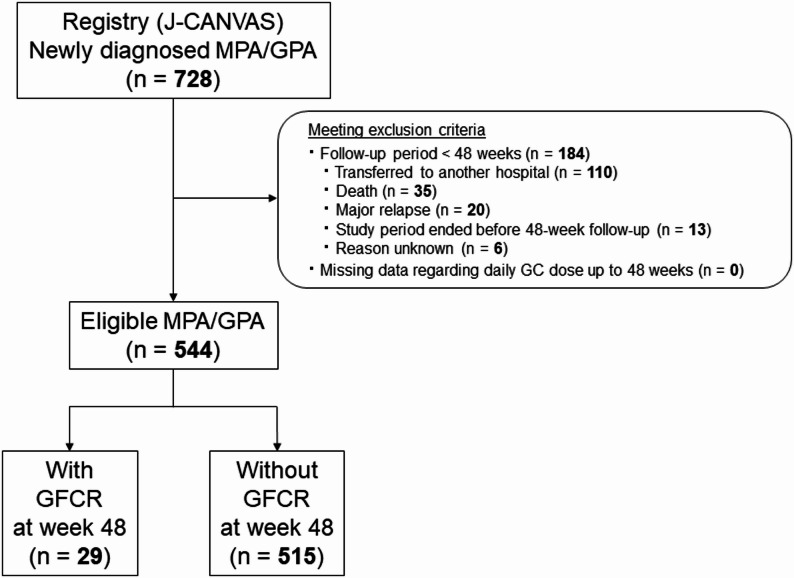
Flowchart of patient selection and stratification according to GFCR status at week 48. *GFCR* Glucocorticoid-Free Clinical Remission, *GPA* Granulomatosis with Polyangiitis, *MPA* Microscopic Polyangiitis

A total of 875 patients were enrolled in the registry during the specified study period, comprising 728 newly diagnosed and 147 relapsing cases of MPA or GPA. The present analysis included the 728 newly diagnosed patients; of these, 184 were excluded from the analysis as they had a follow-up period of < 48 weeks. The reasons for a shorter follow-up were as follows: transfer to another hospital (*n* = 110), death (*n* = 35), major relapse (*n* = 20), study period ended before completion of 48-week follow-up (*n* = 13), and unknown reasons (*n* = 6). Thus, 544 patients were eligible for the analysis (Fig. 1). Compared with the 544 patients with a follow-up of at least 48 weeks, those with < 48 weeks of follow-up tended to be older (79.0 [71.0–83.0] years versus 74.0 [68.0–80.0] years; *p* < 0.001), had higher baseline BVAS (16.0 [12.0–21.0] versus 14.0 [10.0–19.0]; *p* < 0.001), and were more likely to have cardiovascular (9.8% versus 3.1%; *p* = 0.001) or renal involvement (82.6% versus 70.4%; *p* = 0.001), suggesting more severe disease activity and poorer general condition at baseline (Supplementary Table 1).

### GFCR at week 48

Among those with a follow-up of at least 48 weeks, 29 patients (5.3%) achieved GFCR, while 515 patients (94.7%) did not (Fig. 1).

### Patient characteristics and clinical outcomes up to week 48

Baseline characteristics of patients with and without GFCR at 48 weeks are shown in Table 1. There were no significant differences between the two groups in terms of age, sex, vasculitis subtype, ANCA status, comorbidities, BVAS, organ involvement, or laboratory findings at diagnosis (all *p* > 0.05; Table 1).

**Table 1:** Baseline characteristics (at diagnosis) of patients with and without GFCR at week 48

	**All patients (** *n* ** = 544)**	**With GFCR (** *n* ** = 29)**	Without GFCR **(** *n* ** = 515)**	*p*
Age, years	74.0 [68.0–80.0]	74.0 [68.0–79.5]	74.0 [68.0–80.0]	0.740
Sex, Female, n (%)	327 (60.1)	16 (55.2)	311 (60.4)	0.566
Type of vasculitis				
MPA, n (%)	399 (73.3)	24 (82.8)	375 (72.8)	0.286
GPA, n (%)	145 (26.7)	5 (17.2)	140 (27.2)	0.286
ANCA status				
MPO-ANCA positive, n (%)	475 (87.3)	28 (96.6)	447 (86.8)	0.157
PR3-ANCA positive, n (%)	60 (11.0)	1 (3.5)	59 (11.5)	0.235
negative, n (%)	9 (1.7)	0 (0)	9 (1.8)	1.000
Comorbidity				
Hypertension, n (%)	242 (44.5)	14 (48.3)	228 (44.3)	0.704
Diabetes, n (%)	119 (21.9)	5 (17.2)	114 (22.1)	0.649
Chronic kidney disease, n (%)	78 (14.3)	4 (13.8)	74 (14.4)	1.000
Cardiac disease, n (%)	71 (13.1)	2 (6.9)	69 (13.4)	0.407
Cancer, n (%)	37 (6.8)	0 (0)	37 (7.2)	0.249
Birmingham Vasculitis Activity Score (BVAS)	14.0 [10.0–19.0]	14.0 [8.0–20.0]	14.0 [10.0–19.0]	0.889
Organ involvement (BVAS ≥ 1) †				
General, n (%)	373 (68.6)	19 (65.5)	354 (68.7)	0.686
Cutaneous, n (%)	105 (19.3)	7 (24.1)	98 (19.0)	0.473
Mucous membranes or eyes, n (%)	66 (12.1)	3 (10.3)	63 (12.2)	1.000
Ear, nose, and throat, n (%)	157 (28.9)	9 (31.0)	148 (28.7)	0.834
Chest, n (%)	245 (45.0)	15 (51.7)	230 (44.7)	0.566
Cardiovascular, n (%)	17 (3.1)	0 (0)	17 (3.3)	1.000
Abdominal, n (%)	6 (1.1)	0 (0)	6 (1.2)	1.000
Renal, n (%)	383 (70.4)	22 (75.9)	361 (70.1)	0.676
Nervous system, n (%)	146 (26.8)	7 (24.1)	139 (27.0)	0.832
Laboratory data at diagnosis				
S-albumin, mg/dL (*n* = 28, *n* = 505)	2.6 [2.2–3.2]	2.6 [2.0–3.2]	2.6 [2.3–3.2]	0.357
S-creatinine, mg/dL	0.87 [0.64–1.43]	0.89 [0.73–1.72]	0.87 [0.64–1.43]	0.483
eGFR, ml/min/1.73 m^2^	55.5 [32.5–76.4]	54.0 [29.7–75.3]	55.6 [33.4–76.5]	0.608
Hemoglobin, mg/dL (*n* = 29, *n* = 512)	10.4 [9.2–11.8]	10.6 [9.2–12.1]	10.4 [9.2–11.8]	0.451
Neutrophil, /µL (*n* = 29, *n* = 507)	8,331 [5,817–11,920]	9,870 [5,099–11,690]	8,310 [5,840–11,920]	0.886
Lymphocyte, /µL (*n* = 29, *n* = 507)	1,254 [930–1,690]	1,110 [800–1,645]	1,260 [939–1,692]	0.355
Serum IgG, mg/dL (*n* = 27, *n* = 474)	1,627 [1,323–2,024]	1,669 [1,351–2,074]	1,623 [1,320–2,014]	0.714
CRP, mg/dL (*n* = 29, *n* = 513)	8.2 [2.5–13.2]	7.6 [0.6–14.5]	8.2 [2.7–13.1]	0.585

Data are presented as median [IQR] or as n (%), unless otherwise indicated

*ANCA* Antineutrophil Cytoplasmic Antibody, *BVAS* Birmingham Vasculitis Activity Score, *CRP* C-Reactive Protein, *eGFR* Estimated Glomerular Filtration Rate, *GFCR* Glucocorticoid-Free Clinical Remission, *GPA* Granulomatosis with Polyangiitis, *MPA* Microscopic Polyangiitis, *MPO* Anti-Myeloperoxidase, *PR3* Anti-Proteinase 3

For statistical analyses, *p < 0.05, **p < 0.01. *p*-value: Wilcoxon rank sum test, Fisher’s exact test

†Organ involvement was based on BVAS ≥ 1

In terms of treatment regimens and clinical outcomes up to week 48 (Table 2), patients who achieved GFCR by 48 weeks tended to have a lower initial GC dose compared to those who did not (35.0 [30.0–50.0] mg/day versus 40.0 [30.0–50.0] mg/day), but this difference was not statistically significant (*p* = 0.051). Furthermore, the daily GC doses at week 1 and subsequent time points (weeks 2, 4, 8, 12, 16, 20, 24, and 48) were significantly lower in patients who achieved GFCR.

**Table 2:** Treatment details and outcomes (up to week 48) in patients with and without GFCR at week 48

	All patients (*n* = 544)	With GFCR (*n* = 29)	Without GFCR (*n* = 515)	*p*
Year in which remission induction therapy was initiated, *n* (%)				
2017–2018	196 (36.0)	13 (44.8)	183 (35.5)	0.325
2019–2020	182 (33.5)	6 (20.7)	176 (34.2)	0.159
2021–2023	166 (30.5)	10 (34.5)	156 (30.3)	0.680
Daily GC dose (prednisolone-equivalent) at each time point				
Initial dose, mg	40.0 [30.0–50.0]	35.0 [30.0–50.0]	40.0 [30.0–50.0]	0.051
Initial dose, mg/kg (*n* = 28, *n* = 513)	0.82 [0.64–0.98]	0.69 [0.58–0.97]	0.83 [0.64–0.98]	0.123
at week 1, mg	40.0 [30.0–50.0]	30.0 [27.5–50.0]	40.0 [30.0–50.0]	0.048^*^
at week 2, mg	40.0 [30.0–45.0]	30.0 [15.0–40.0]	40.0 [30.0–45.0]	0.001^**^
at week 4, mg	30.0 [25.0–40.0]	20.0 [7.5–30.0]	30.0 [25.0–40.0]	< 0.001^**^
at week 8, mg	20.0 [17.0–27.0]	15.0 [4.0–20.0]	20.0 [17.5–27.0]	< 0.001^**^
at week 12, mg	17.5 [12.5–20.0]	7.5 [2.0–15.0]	17.5 [12.5–20.0]	< 0.001^**^
at week 16, mg	15.0 [10.0–17.5]	5.0 [0–10.0]	15.0 [10.0–17.5]	< 0.001^**^
at week 20, mg	12.5 [9.0–15.0]	2.5 [0–7.3]	12.5 [10.0–15.0]	< 0.001^**^
at week 24, mg	10.0 [8.0–14.0]	1.0 [0–5.0]	10.0 [8.0–14.0]	< 0.001^**^
at week 48, mg	7.0 [5.0–9.0]	0 [0–0]	7.0 [5.0–10.0]	< 0.001^**^
Treatment up to week 24				
Induction therapy (RTX/IVCYC)				
Both RTX and IVCYC, n (%)	19 (3.5)	0 (0)	19 (3.7)	0.616
RTX without IVCYC, n (%)	142 (26.1)	18 (62.1)	124 (24.1)	< 0.001^**^
IVCYC without RTX, n (%)	198 (36.4)	4 (13.8)	194 (37.7)	0.009^**^
Neither RTX nor IVCYC, n (%)	185 (34.0)	7 (24.1)	178 (34.6)	0.315
Other immunosuppressive agents				
AZA, n (%)	182 (33.5)	5 (17.2)	177 (34.4)	0.068
MMF, n (%)	11 (2.0)	0 (0)	11 (2.1)	1.000
MTX, n (%)	20 (3.7)	1 (3.5)	19 (3.7)	1.000
MZR, n (%)	20 (3.7)	2 (6.9)	18 (3.5)	0.289
Adjunctive therapy				
Methylprednisolone pulse, n (%)	177 (32.5)	2 (6.9)	175 (34.0)	0.002^**^
PLEX, n (%)	29 (5.3)	0 (0)	29 (5.6)	0.391
Avacopan, n (%)	15 (2.8)	6 (20.7)	9 (1.8)	< 0.001^**^
Treatment from weeks 24–48				
Maintenance therapy				
RTX, n (%)	71 (13.1)	7 (24.1)	64 (12.4)	0.085
AZA, n (%)	222 (40.8)	7 (24.1)	215 (41.8)	0.079
MMF, n (%)	24 (4.4)	1 (3.5)	23 (4.5)	1.000
MTX, n (%)	29 (5.3)	1 (3.5)	28 (5.4)	1.000
MZR, n (%)	30 (5.5)	1 (3.5)	29 (5.6)	1.000
Adjunctive therapy				
Avacopan, n (%)	10 (1.8)	4 (13.8)	6 (1.2)	0.001^**^
Outcomes up to week 48				
Death, n (%)	0 (0)	0 (0)	0 (0)	-
Major relapse, n (%)	0 (0)	0 (0)	0 (0)	-
Minor relapse, n (%)	31 (5.7)	0 (0)	31 (6.0)	0.399
Severe infection, n (%)	41 (7.5)	0 (0)	41 (8.0)	0.155
Renal outcomes through week 48 among patients with renal involvement at baseline (all patients, *n* = 383; with GFCR, *n* = 22; without GFCR, *n* = 361) †				
eGFR at baseline, ml/min/1.73 m^2^ (*n* = 22, *n* = 361)	44.3 [24.4–65.2]	37.2 [23.6–65.1]	45.0 [24.5–65.4]	0.737
eGFR at week 4, ml/min/1.73 m^2^ (*n* = 22, *n* = 354)	44.3 [29.9–63.1]	40.7 [29.1–64.1]	44.4 [30.0–63.2]	0.916
eGFR at week 12, ml/min/1.73 m^2^ (*n* = 22, *n* = 356)	46.0 [32.9–62.2]	44.2 [32.8–56.8]	46.2 [32.8–62.5]	0.838
eGFR at week 24, ml/min/1.73 m^2^ (*n* = 22, *n* = 357)	44.6 [32.7–59.5]	41.5 [31.6–60.3]	44.8 [32.9–59.4]	0.682
eGFR at week 48, ml/min/1.73 m^2^ (*n* = 22, *n* = 355)	44.8 [32.7–57.1]	45.9 [36.3–56.1]	44.7 [32.0–57.4]	0.692
Changes in eGFR from baseline to week 48, ml/min/1.73 m² (*n* = 22, *n* = 355)	−0.2 [− 11.3–9.1]	2.0 [− 6.1–10.9]	−0.4 [− 11.8–9.0]	0.208
Progression to end-stage kidney disease, n (%) ‡	16 (4.2)	1 (4.6)	15 (4.2)	1.000

Data are presented as median [IQR] or as n (%), unless otherwise indicated

*AZA* Azathioprine, *eGFR* Estimated Glomerular Filtration Rate, *GC* Glucocorticoid, *GFCR* Glucocorticoid-Free Clinical Remission, *IVCYC* Intravenous Cyclophosphamide, *MMF* Mycophenolate Mofetil, *MTX* Methotrexate, *MZR* Mizoribine, *PLEX* Plasma Exchange, *RTX* Rituximab

For statistical analyses, *p < 0.05, **p < 0.01. *p*-value: Wilcoxon rank sum test, Fisher’s exact test

† Renal involvement was defined as BVAS renal item ≥ 1

‡End-stage kidney disease was defined as an eGFR < 15 mL/min/1.73 m² or permanent kidney replacement therapy (hemodialysis or peritoneal dialysis)

Regarding concomitant therapies within the first 24 weeks (Table 2), patients who achieved GFCR were significantly more likely to receive RTX without intravenous cyclophosphamide (IVCYC) (62.1% versus 24.1%; *p* < 0.001) and avacopan (20.7% versus 1.8%; *p* < 0.001). In contrast, these patients were significantly less likely to have received IVCYC without RTX (13.8% versus 37.7%; *p* = 0.009) or methylprednisolone pulse therapy (6.9% versus 34.0%; *p* = 0.002). The dosing of RTX and IVCYC per course was not significantly different between groups, but patients who achieved GFCR received RTX more frequently during induction and maintenance phases (Supplementary Table 2). The use of avacopan between weeks 24 and 48 was also more common among those who achieved GFCR (13.8% versus 1.2%; *p* = 0.001; Table 2).

The incidence of minor relapse and serious infection up to week 48 was comparable between groups. Among patients with renal involvement at baseline (*n* = 383), there were no significant differences between groups in terms of median eGFR at weeks 4, 12, 24, and 48, as well as changes in eGFR from baseline to week 48. Progression to end-stage kidney disease within 48 weeks was infrequent and comparable between both groups (Table 2).

### Predictive factors associated with GFCR at 48 weeks

In the univariable regression analysis, the use of RTX for induction therapy and the use of avacopan within 48 weeks were found to be significantly associated with a higher likelihood of achieving GFCR at week 48 (RTX – OR: 5.16, 95% CI: 2.37–11.22, *p* < 0.001; avacopan – OR: 14.7, 95% CI: 4.8–44.7, *p* < 0.001). Conversely, the use of IVCYC and methylprednisolone pulse therapy was significantly associated with a lower likelihood of achieving GFCR (IVCYC – OR: 0.26, 95% CI: 0.09–0.77, *p* = 0.015; methylprednisolone pulse – OR: 0.14, 95% CI: 0.03–0.61, *p* = 0.009). Other demographic and clinical characteristics at baseline, including AAV subtype, ANCA status, BVAS, organ involvement, and laboratory parameters, were not significantly associated with GFCR (Table 3).

**Table 3:** Results of univariable and multivariable logistic regression analyses for predictors of GFCR at week 48

Factors	Univariable analysis		Multivariable analysis, model 1†		Multivariable analysis, model 2‡	
	OR (95% CI)	*p*	OR (95% CI)	*p*	OR (95% CI)	*p*
Age	1.00 (0.97–1.03)	0.798				
Female	0.81 (0.38–1.71)	0.577				
MPA	1.79 (0.67–4.79)	0.245				
MPO-ANCA positive	4.26 (0.57–31.82)	0.158			3.82 (0.49–29.60)	0.199
BVAS	1.01 (0.96–1.06)	0.758				
Organ involvement (BVAS ≥ 1) †						
General	0.86 (0.39–1.90)	0.716				
Cutaneous	1.35 (0.56–3.26)	0.499				
Mucous Membranes or eyes	0.83 (0.24–2.81)	0.762				
Ear, nose, and throat	1.12 (0.50–2.51)	0.791				
Chest	1.33 (0.63–2.81)	0.458				
Renal	1.34 (0.56–3.20)	0.510				
Nervous system	0.86 (0.36–2.06)	0.736				
Laboratory data at diagnosis						
S-albumin, mg/dL	0.82 (0.46–1.46)	0.499				
eGFR, ml/min/1.73 m^2^	1.00 (0.98–1.01)	0.582				
CRP, mg/dL	0.98 (0.93–1.04)	0.545				
Induction therapy up to 24 weeks						
RTX without IVCYC	5.16 (2.37–11.22)	< 0.001^**^	3.90 (1.52–10.00)	0.005^**^	3.47 (1.25–9.63)	0.017^*^
IVCYC without RTX	0.26 (0.09–0.77)	0.015^*^	0.61 (0.16–2.28)	0.462	0.66 (0.17–2.55)	0.548
Neither RTX nor IVCYC	0.60 (0.25–1.44)	0.253				
Maintenance therapy from 24 to 48 weeks						
RTX	2.24 (0.92–5.46)	0.075			1.18 (0.39–3.54)	0.768
AZA	0.44 (0.19–1.06)	0.067			0.67 (0.24–1.86)	0.446
Adjunctive therapy						
Use of methylprednisolone pulse up to 24 weeks	0.14 (0.03–0.61)	0.009^**^	0.08 (0.02–0.44)	0.004^**^	0.08 (0.02–0.44)	0.004^**^
Use of avacopan up to 48 weeks	14.7 (4.8–44.7)	< 0.001^**^	24.3 (5.8–101.9)	< 0.001^**^	22.9 (5.3–98.1)	< 0.001^**^

Logistic regression models were applied for univariable and multivariable analysis

†p-values are from model 1 of multivariable logistic regression including factors with p-values < 0.05 in univariable analysis

‡p-values are from model 2 of multivariable logistic regression including factors with p-values < 0.20 in univariable analysis

*ANCA* Antineutrophil Cytoplasmic Antibody, *AZA* Azathioprine, *BVAS* Birmingham Vasculitis Activity Score, *CRP* C-Reactive Protein, *eGFR* Estimated Glomerular Filtration Rate, *IVCYC* Intravenous Cyclophosphamide, *MPA* Microscopic Polyangiitis, *MPO* Anti-Myeloperoxidase, *RTX* Rituximab

For statistical analyses, *p < 0.05, **p < 0.01

Based on these results, the factors—use of RTX for induction therapy, avacopan, IVCYC, and methylprednisolone pulse therapy—were subjected to multivariable logistic regression analysis. The analysis revealed that the use of RTX for induction therapy and avacopan within 48 weeks was independently and significantly associated with achieving GFCR. In model 1, RTX use was associated with GFCR with an OR of 3.90 (95% CI: 1.52–10.00, *p* = 0.005), and avacopan use with an OR of 24.3 (95% CI: 5.8–101.9, *p* < 0.001). These associations remained robust in model 2 (RTX – OR: 3.47, 95% CI: 1.25–9.63, *p* = 0.017; avacopan – OR: 22.9, 95% CI: 5.3–98.1, *p* < 0.001). Methylprednisolone pulse therapy was also independently associated with a significantly lower likelihood of achieving GFCR in both models (model 1: OR: 0.08, 95% CI: 0.02–0.44, *p* = 0.004; model 2: OR: 0.08, 95% CI: 0.02–0.44, *p* = 0.004). IVCYC use, although significant in the univariable analysis, did not show a statistically significant association in the multivariable models (Table 3).

**Fig. 2:**
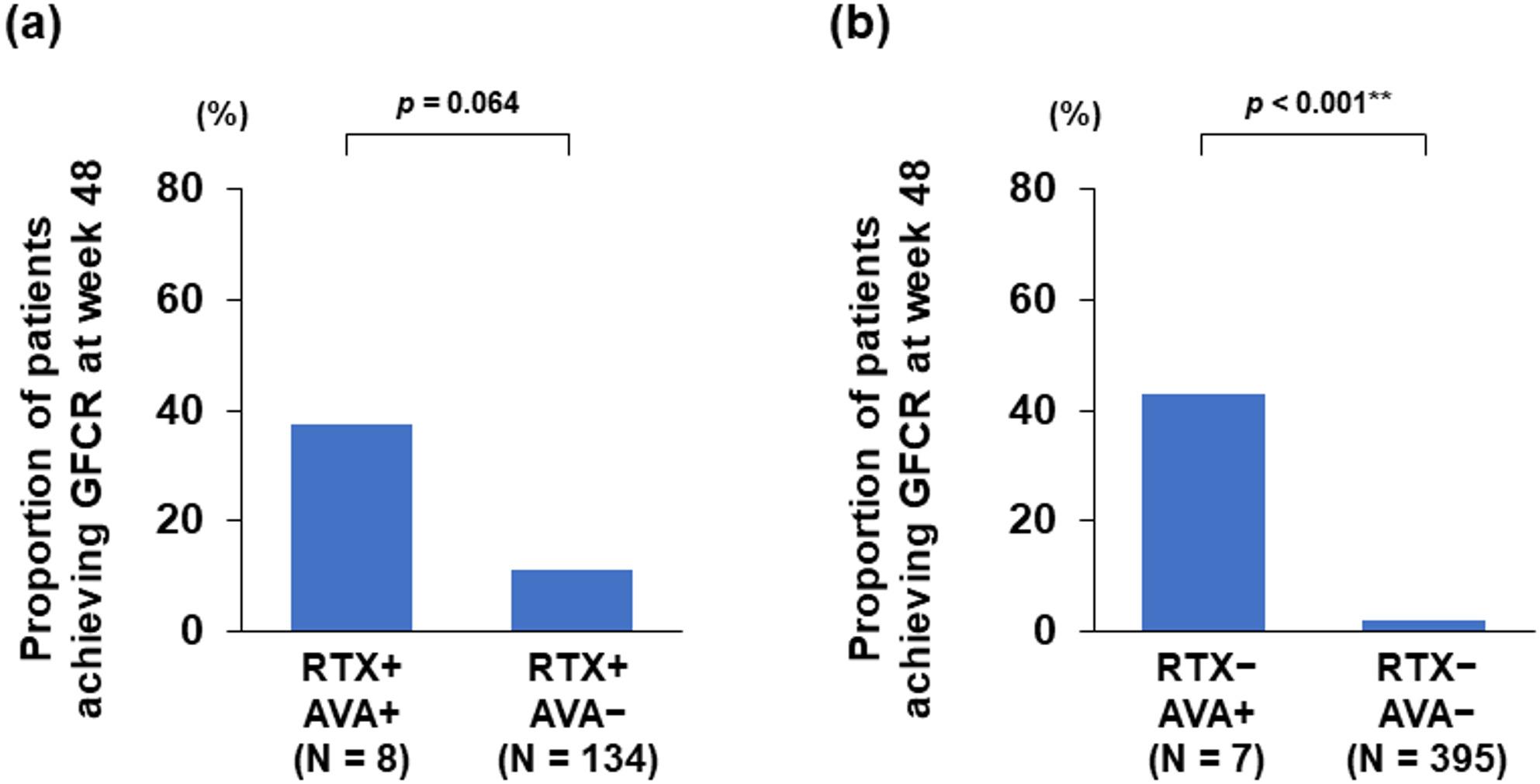
Distribution pattern of GFCR at week 48 in study patients stratified by RTX induction therapy and avacopan use. **a** Patients who received RTX for induction therapy (*n* = 142) and **b** patients who did not (*n* = 402). Bars represent the proportion of patients achieving GFCR in each treatment group, defined by RTX use for induction therapy and avacopan use within 48 weeks: RTX + AVA+ (both agents), RTX + AVA− (RTX alone), RTX – AVA + (avacopan alone), and RTX – AVA − (neither agent). *AVA* Avacopan, *GFCR* Glucocorticoid-Free Clinical Remission, *RTX* Rituximab. For statistical analyses, *p < 0.05, **p < 0.01. *p*-value: Fisher’s exact test

Figure 2 illustrates the distribution of patients achieving GFCR according to the use of RTX and avacopan. In patients who received RTX for induction therapy (Fig. 2a), GFCR was achieved in 37.5% of those who also received avacopan and in 12.7% of those who did not receive avacopan. Conversely, in patients who did not receive RTX (Fig. 2b), GFCR was achieved in 42.9% of those who received avacopan and in 2.0% of those who did not. These findings indicate that the use of avacopan was associated with higher GFCR rates regardless of RTX use, consistent with the associations identified in the regression analyses.

To further validate these findings, a sensitivity analysis was performed using a matched cohort of 28 patients with GFCR and 81 without GFCR. Baseline characteristics were well balanced between the two groups (Supplementary Table 3). Consistent with the overall cohort, patients in the GFCR group of the matched cohort also had significantly lower cumulative GC doses through week 48, were more likely to have received RTX and avacopan, and were less likely to have received methylprednisolone pulse therapy (Supplementary Table 4). Likewise, in the matched cohort, both RTX and avacopan use were significantly associated with achieving GFCR in both the univariable and multivariable analyses, reinforcing the robustness of the main findings (Supplementary Table 5).

To explore potential residual confounding related to the choice of induction therapy, an exploratory restricted-cohort comparison was conducted between patients treated with RTX without IVCYC versus those treated with IVCYC without RTX. Patients who received both RTX and IVCYC, neither RTX nor IVCYC, methylprednisolone pulse therapy, PLEX, or avacopan were excluded. The two groups had broadly comparable baseline characteristics, although differences remained in the calendar year of induction therapy initiation and GC tapering patterns between groups (Supplementary Tables 6 and 7). This suggests the presence of residual confounding related to evolving treatment strategies.

### Long-term clinical outcomes between weeks 48 and 96

**Fig. 3:**
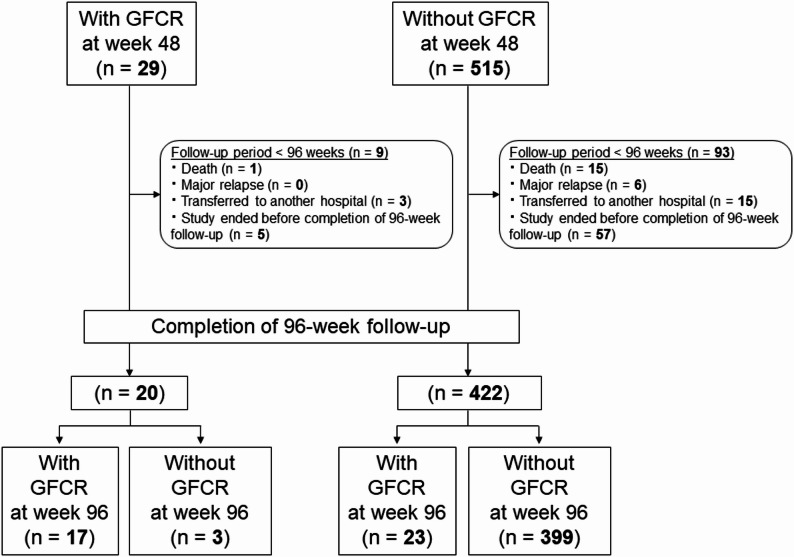
Flowchart of patients according to GFCR status at week 48 and long-term clinical outcomes during weeks 48–96. *GFCR* Glucocorticoid-Free Clinical Remission

In the main cohort, the rates of death and major relapse between weeks 48 and 96 were comparable between patients who did or did not achieve GFCR by week 48 (death: 3.5% versus 2.9%, *p* = 0.589; major relapse: 0% versus 1.2%, *p* = 1.000). However, patients who had achieved GFCR at week 48 were significantly more likely to maintain GFCR at week 96 compared to those who had not (58.6% versus 4.5%, *p* < 0.001). No other statistically significant differences were observed in the incidence of minor relapse or serious infections during this period (Fig. 3; Table 4). All serious infections occurred in patients who did not achieve GFCR; however, this finding did not reach statistical significance.

**Table 4:** Long-term (weeks 48–96) clinical outcomes of study patients with and without GFCR at week 48

	**All patients (** *n* ** = 544)**	With GFCR **(** *n* ** = 29)**	Without GFCR **(** *n* ** = 515)**	*p*
Discontinuation of follow-up between weeks 48 and 96	102 (18.8)	9 (31.0)	93 (18.1)	0.089
Death, n (%)	16 (2.9)	1 (3.5)	15 (2.9)	0.589
of which, due to vasculitis-worsening or infections, n (%)	8 (1.6)	0 (0)	8 (1.6)	1.000
Major relapse, n (%)	6 (1.1)	0 (0)	6 (1.2)	1.000
Lost to follow-up due to transfer, n (%)	18 (3.3)	3 (10.3)	15 (2.9)	0.065
Study period ended before completion of the 96-week follow-up, n (%)	62 (11.4)	5 (17.2)	57 (11.1)	0.360
Completed 96-week follow-up	442 (81.3)	20 (69.0)	422 (81.9)	0.089
GFCR at week 96	40 (7.4)	17 (58.6)	23 (4.5)	< 0.001^**^
Daily GC dose at week 96, mg (*N* = 20, *N* = 422)	5.0 [3.0–7.0]	0 [0–0]	5.0 [4.0–7.0]	< 0.001^**^
Minor relapse between weeks 48 and 96, n (%)	30 (5.5)	1 (3.5)	29 (5.6)	1.000
Serious infection between weeks 48 and 96, n (%)	19 (3.5)	0 (0)	19 (3.7)	0.616
Treatment between weeks 48 and 96				
RTX (maintenance), n (%)	80 (14.7)	11 (37.9)	69 (13.4)	0.001^**^
AZA, n (%)	197 (36.2)	7 (24.1)	190 (36.9)	0.233
MMF, n (%)	30 (5.5)	1 (3.5)	29 (5.6)	1.000
MTX, n (%)	32 (5.9)	1 (3.5)	31 (6.0)	1.000
MZR, n (%)	37 (6.8)	1 (3.5)	36 (7.0)	0.712
Avacopan, n (%) †	20 (3.7)	4 (13.8)	16 (3.1)	0.017^*^

Data are presented as median [IQR] or as n (%), unless otherwise indicated

*AZA* Azathioprine, *GFCR* Glucocorticoid-Free Clinical Remission, *MMF* Mycophenolate Mofetil, *MTX* Methotrexate, *MZR* Mizoribine, *RTX* Rituximab

For statistical analyses, *p < 0.05, **p < 0.01. *p*-value: Wilcoxon rank sum test or Fisher’s exact test

† Among patients who received avacopan between weeks 48 and 96, avacopan was continued beyond week 48 in all 4 patients with GFCR and in 6 out of 16 patients without GFCR. Among those who continued avacopan, the median duration of treatment was 90.6 [61.0–93.6] weeks in patients with GFCR and 91.8 [75.8–95.4] weeks in those without GFCR

Maintenance therapies notably differed between groups. Patients who achieved GFCR at week 48 were more likely to receive RTX between weeks 48 and 96 (37.9% versus 13.4%, *p* = 0.001), and avacopan use during this period was also more frequent in the GFCR group (13.8% versus 3.1%, *p* = 0.017) (Table 4). Overall, only a few patients used avacopan between weeks 48 and 96, and this was continued beyond week 48 in all 4 patients with GFCR and in 6 out of 16 patients without GFCR, reflecting physician discretion in routine clinical practice. Among patients who continued avacopan beyond week 48, the median duration of treatment was approximately 90 weeks in both groups (Table 4).

Regarding long-term outcomes in the matched cohort, follow-up discontinuation between weeks 48 and 96 was more frequent in the GFCR group (32.1% versus 12.4%, *p* = 0.039); however, the incidence of death or major relapse did not vary significantly between the two groups (death: 3.6% versus 0%, *p* = 0.257; major relapse: 0% versus 0%). Consistent with the findings of the overall cohort, patients who had achieved GFCR at week 48 were significantly more likely to maintain GFCR at week 96 compared to those who had not (57.1% versus 4.9%, *p* < 0.001). The incidence of minor relapse and serious infections was also similar between the two groups (Supplementary Table 8).

## Discussion

This multicenter study evaluated the rate of GFCR 48 weeks after treatment initiation, factors associated with achieving GFCR, and long-term clinical outcomes between weeks 48 and 96 in patients with newly diagnosed MPA and GPA. We observed that only 5.3% of patients achieved GFCR at week 48, highlighting the complexities hampering complete GC withdrawal within the first year of treatment. Unlike randomized controlled trials that have standardized protocols, GC tapering and discontinuation in clinical practice are individualized, and complete discontinuation within 1 year is not uniformly pursued, which may partly explain the low GFCR rate observed in our cohort despite the low relapse rates. The low GFCR rates also reflect the challenges in balancing effective disease suppression with curtailing GC exposure in clinical practice. Our findings provide important insights into treatment strategies to facilitate early GC tapering and sustained disease control in clinical settings.

In the multivariable analysis, both induction therapy with RTX and the use of avacopan within 48 weeks were independently associated with achieving GFCR at week 48. To date, two pivotal randomized controlled trials—RAVE [16] and RITUXVAS [17]—have compared RTX use with IVCYC as induction therapy in AAV. These studies demonstrated that RTX was non-inferior to IVCYC in terms of achieving remission at both 6 and 12 months post-induction; however, because both arms of these trials followed the same GC tapering protocol, it remains unclear whether RTX facilitates GC tapering more effectively than IVCYC. More recently, the LoVAS [10] and RITAZAREM [18] trials demonstrated that RTX use with a reduced-dose GC regimen was comparable to RTX with a high-dose GC regimen in terms of remission rates, supporting the use of RTX as an effective induction agent even when combined with reduced GC regimens. In addition, avacopan, a selective C5a receptor antagonist, offers a GC-sparing approach to inflammation control by blocking complement-mediated neutrophil activation [19, 20]. In the ADVOCATE trial [11], avacopan was shown to be non-inferior to a standard-dose prednisolone for inducing remission at week 26 and superior for maintaining sustained remission at week 52. Our findings corroborate the GC-sparing efficacy of RTX and avacopan in clinical settings, but this association should be interpreted cautiously. In our exploratory analysis comparing patients treated with RTX without IVCYC versus those treated with IVCYC without RTX, there remained differences in calendar year of treatment initiation, suggesting that recent treatment strategies focusing on rapid GC tapering may have contributed to the observed association (Supplementary Tables 6 and 7). The rate of GC tapering was slower in patients who achieved GFCR versus those in the reduced-dose GC arm of the LoVAS trial [10], which could be attributed to the inclusion of patients with severe organ-threatening involvement and the absence of protocolized GC tapering in routine clinical practice. Notably, no demographic or disease-related variables, including age, sex, AAV subtype, ANCA status, BVAS, and organ involvement, were found to predict the achievement of GFCR. This finding highlights how treatment strategy is more crucial than baseline disease characteristics in achieving successful GC withdrawal.

In contrast, methylprednisolone pulse therapy was found to be an independent negative predictor of achieving GFCR at week 48. Patients who received methylprednisolone pulse therapy had higher BVAS, more pulmonary and renal involvement, and worse renal function at diagnosis (Supplementary Table 9). Furthermore, these patients were more likely to require intensive therapies, such as IVCYC or PLEX, and underwent slower GC tapering (Supplementary Table 10). The association between methylprednisolone pulse therapy use and a lower likelihood of GFCR may partly reflect confounding by indication, in which methylprednisolone pulse therapy is preferentially used for patients with more severe disease, along with other treatment protocols that prolong GC exposure. This negative association persisted even in the matched cohort even after balancing BVAS and other key baseline characteristics. This suggests the difficulty of fully adjusting for acute, organ-threatening features that prompt pulse therapy, many of which were not entirely captured by matching variables, resulting in residual confounding.

Although not identified as an independent predictor on multivariable analysis, treatment during the maintenance phase (weeks 24–48) may also influence GFCR at week 48. In our study, patients who achieved GFCR more frequently received RTX as maintenance therapy and less frequently received azathioprine. Although these differences were not statistically significant, this trend favoring RTX over azathioprine for maintenance therapy is consistent with previous randomized controlled trials. Both the MAINRITSAN and RITAZAREM trials [21, 22] demonstrated lower relapse rates with RTX compared to azathioprine in the maintenance phase under standardized GC tapering. Furthermore, a pooled analysis from the MAINRITSAN studies [23] showed that patients treated with RTX had significantly lower cumulative GC doses, suggesting that RTX better supports GC tapering during the maintenance phase and can facilitate transition to GFCR status. Likewise, GFCR at week 96 may be influenced by maintenance therapy during weeks 48–96. In our cohort, patients who achieved GFCR at week 48 were more likely to receive RTX and avacopan between weeks 48 and 96, suggesting that sustained GFCR may have been influenced by more intensive maintenance strategies. Therefore, while early GFCR at week 48 primarily reflects the effectiveness of induction therapies, maintenance therapy likely contributed to long-term GFCR beyond 1 year.

Overall, our results highlight the importance of individualized immunosuppressive strategies incorporating RTX and avacopan to enable earlier and sustained GC tapering without compromising disease control. Furthermore, despite the small number of events, no serious infections occurred among patients who achieved GFCR during follow-up, underlining the importance of minimizing GC exposure. However, this study has several limitations. First, its retrospective design introduces potential bias due to unmeasured confounding, including indication bias in treatment selection. This is because treatment strategies, such as the choice and timing of immunosuppressive agents and GC tapering schedules, were not standardized across centers. In addition, avacopan use itself may have influenced physicians’ willingness to pursue more proactive GC tapering, potentially contributing to the higher GFCR rates independent of the pharmacologic effect of avacopan. Second, the number of patients who achieved GFCR was relatively small (5.3% of the overall cohort), limiting the statistical power of our results to identify additional predictors. To address this, we performed a sensitivity analysis using a matched cohort based on key baseline characteristics; the analysis corroborated our results from the main cohort, supporting the robustness of our findings. Third, patients with < 48 weeks of follow-up were excluded from the main analysis; it is noteworthy that these patients tended to have more severe disease activity and worse general condition at baseline, which may have introduced selection bias. However, because the primary outcome was defined as GFCR at week 48, their exclusion was methodologically necessary. Additional analysis including these patients has been provided for transparency (Supplementary Table 1). Fourth, since the analyses focused on early GFCR at week 48, we did not evaluate the predictors of longer-term GFCR beyond 1 year, which may be more strongly influenced by maintenance therapy. Finally, although it was a multicenter study, the patient population was based in Japan, limiting the generalizability of our findings to populations with similar demographic and disease characteristics. Prospective validation in broader cohorts is warranted.

## Conclusions

Our multicenter cohort study identified the use of RTX for induction therapy and the use of avacopan within 48 weeks of treatment initiation as independent predictors of achieving GFCR at week 48 in patients with newly diagnosed MPA or GPA. These findings support the feasibility of GC-sparing strategies in AAV and highlight the potential of RTX and avacopan as key treatment components facilitating GFCR. Future prospective studies are needed to validate these results and optimize treatment algorithms for achieving disease control while minimizing GC-related toxicity.

## Supplementary Information

Supplementary Material 1.

Supplementary Material 2.

Supplementary Material 3.

Supplementary Material 4.

Supplementary Material 5.

Supplementary Material 6.

Supplementary Material 7.

Supplementary Material 8.

Supplementary Material 9.

Supplementary Material 10.

## Data Availability

The datasets used and/or analyzed in this study can be obtained from the corresponding author upon reasonable request.

## Abbreviations

ANCA: Antineutrophil cytoplasmic antibody
AAV: ANCA-associated vasculitis
MPA: Microscopic polyangiitis
GPA: Granulomatosis with polyangiitis
EGPA: Eosinophilic granulomatosis with polyangiitis
GC: Glucocorticoid
RTX: Rituximab
CYC: Cyclophosphamide
GFCR: GC-free clinical remission
J-CANVAS: The Japan Collaborative Registry of ANCA-Associated Vasculitis
CRP: C-reactive protein
MPO: Myeloperoxidase
PR3: Proteinase 3
BVAS: Birmingham Vasculitis Activity Score
PLEX: Plasma exchange
CR: Clinical remission
IVCYC: Intravenous cyclophosphamide

## References

[1] Jennette JC, Falk RJ, Bacon PA, Basu N, Cid MC, Ferrario F, et al. 2012 revised international chapel hill consensus conference nomenclature of vasculitides. Arthritis Rheum. 2013;65:1–1. 10.1002/art.37715 23045170

[2] Wallace ZS, Miloslavsky EM. Management of ANCA associated vasculitis. BMJ. 2020;18:m421. 10.1136/bmj.m421 32188597

[3] Kidney Disease: Improving Global Outcomes (KDIGO) ANCA Vasculitis Work Group. KDIGO 2024 clinical practice guideline for the management of antineutrophil cytoplasmic antibody (ANCA)-associated vasculitis. Kidney Int. 2024;105:S71–116. 38388102 10.1016/j.kint.2023.10.008

[4] Hellmich B, Sanchez-Alamo B, Schirmer JH, Berti A, Blockmans D, Cid MC, et al. EULAR recommendations for the management of ANCA-associated vasculitis: 2022 update. Ann Rheum Dis. 2024;83:30–47. 36927642 10.1136/ard-2022-223764

[5] Chung SA, Langford CA, Maz M, Abril A, Gorelik M, Guyatt G, et al. 2021 American College of Rheumatology/Vasculitis Foundation guideline for the management of antineutrophil cytoplasmic antibody–associated vasculitis. Arthritis Rheumatol. 2021;73:1366–83. 34235894 10.1002/art.41773 PMC12327957

[6] Wall N, Harper L. Complications of long-term therapy for ANCA-associated systemic vasculitis. Nat Rev Nephrol. 2012;8:523–32. 22664736 10.1038/nrneph.2012.107

[7] Robson J, Doll H, Suppiah R, Flossmann O, Harper L, Höglund P, et al. Damage in the anca-associated vasculitides: long-term data from the European vasculitis study group (EUVAS) therapeutic trials. Ann Rheum Dis. 2015;74:177–84. 24243925 10.1136/annrheumdis-2013-203927

[8] Smith R. Complications of therapy for ANCA-associated vasculitis. Rheumatology. 2020;59:iii74–8. 31967652 10.1093/rheumatology/kez618

[9] Walsh M, Merkel PA, Peh CA, Szpirt WM, Puéchal X, Fujimoto S, et al. Plasma Exchange and glucocorticoids in severe ANCA-associated vasculitis. N Engl J Med. 2020;382:622–31. 32053298 10.1056/NEJMoa1803537 PMC7325726

[10] Furuta S, Nakagomi D, Kobayashi Y, Hiraguri M, Sugiyama T, Amano K, et al. Effect of reduced-dose vs high-dose glucocorticoids added to rituximab on remission induction in ANCA-associated vasculitis: a randomized clinical trial. JAMA. 2021;325:2178–87. 34061144 10.1001/jama.2021.6615 PMC8170547

[11] Jayne DRW, Merkel PA, Schall TJ, Bekker P, ADVOCATE Study Group. Avacopan for the treatment of ANCA-associated vasculitis. N Engl J Med. 2021;384:599–609. 33596356 10.1056/NEJMoa2023386

[12] Watts R, Lane S, Hanslik T, Hauser T, Hellmich B, Koldingsnes W, et al. Development and validation of a consensus methodology for the classification of the ANCA-associated vasculitides and polyarteritis nodosa for epidemiological studies. Ann Rheum Dis. 2007;66:222–7. 16901958 10.1136/ard.2006.054593 PMC1798520

[13] Mukhtyar C, Lee R, Brown D, Carruthers D, Dasgupta B, Dubey S, et al. Modification and validation of the Birmingham Vasculitis Activity Score (version 3). Ann Rheum Dis. 2009;68:1827–32. 19054820 10.1136/ard.2008.101279

[14] Yates M, Watts RA, Bajema IM, Cid MC, Crestani B, Hauser T, et al. EULAR/ERA-EDTA recommendations for the management of ANCA-associated vasculitis. Ann Rheum Dis. 2016;75:1583–94. 27338776 10.1136/annrheumdis-2016-209133

[15] Common Terminology Criteria for Adverse Events (CTCAE) version 5. US department of health and human services, national institutes of health, national cancer institute. 2017. https://ctep.cancer.gov/protocolDevelopment/electronic_applications/ctc.htm#ctc_50. Accessed 15 Aug 2025.

[16] Stone JH, Merkel PA, Spiera R, Seo P, Langford CA, Hoffman GS, et al. Rituximab versus cyclophosphamide for ANCA-associated vasculitis. N Engl J Med. 2010;363:221–32. 20647199 10.1056/NEJMoa0909905 PMC3137658

[17] Jones RB, Tervaert JW, Hauser T, Luqmani R, Morgan MD, Peh CA, et al. Rituximab versus cyclophosphamide in ANCA-associated renal vasculitis. N Engl J Med. 2010;363:211–20. 20647198 10.1056/NEJMoa0909169

[18] Smith RM, Jones RB, Specks U, Bond S, Nodale M, Aljayyousi R, et al. Rituximab as therapy to induce remission after relapse in ANCA-associated vasculitis. Ann Rheum Dis. 2020;79:1243–9. 32581088 10.1136/annrheumdis-2019-216863 PMC7456549

[19] Bekker P, Dairaghi D, Seitz L, Leleti M, Wang Y, Ertl L, et al. Characterization of pharmacologic and pharmacokinetic properties of ccx168, a potent and selective orally administered complement 5a receptor inhibitor, based on preclinical evaluation and randomized phase 1 clinical study. PLoS ONE. 2016;11:e0164646. 27768695 10.1371/journal.pone.0164646 PMC5074546

[20] Xiao H, Dairaghi DJ, Powers JP, Ertl LS, Baumgart T, Wang Y, et al. C5a receptor (CD88) blockade protects against MPO-ANCA GN. J Am Soc Nephrol. 2014;25:225–31. 24179165 10.1681/ASN.2013020143 PMC3904560

[21] Guillevin L, Pagnoux C, Karras A, Khouatra C, Aumaître O, Cohen P, et al. Rituximab versus azathioprine for maintenance in ANCA-associated vasculitis. N Engl J Med. 2014;371:1771–80. 25372085 10.1056/NEJMoa1404231

[22] Smith RM, Jones RB, Specks U, Bond S, Nodale M, Al-Jayyousi R, et al. Rituximab versus azathioprine for maintenance of remission for patients with ANCA-associated vasculitis and relapsing disease: an international randomised controlled trial. Ann Rheum Dis. 2023;82:937–44. 36958796 10.1136/ard-2022-223559 PMC10313987

[23] Delestre F, Charles P, Karras A, Pagnoux C, Néel A, Cohen P, et al. Rituximab as maintenance therapy for ANCA-associated vasculitides: pooled analysis and long-term outcome of 277 patients included in the MAINRITSAN trials. Ann Rheum Dis. 2024;83:233–41. 37918894 10.1136/ard-2023-224623

