# Supplementary material for: Predictors of glucocorticoid-free clinical remission in patients with newly diagnosed microscopic polyangiitis and granulomatosis with polyangiitis: a retrospective cohort study using a nationwide registry in Japan (J-CANVAS)
Source: Arthritis Res Ther. 2026 Mar 10;28:89. doi: 10.1186/s13075-026-03780-3 (PMC13085565; doi:10.1186/s13075-026-03780-3)
Supplement: Supplementary file 1 — Supplementary Material 1. [file 13075_2026_3780_MOESM1_ESM.docx]

Supplementary Table 1. Baseline characteristics (at diagnosis) of study patients with and without 48-week follow-up

|  | All patients (n = 728) | Follow-up period  ≥ 48 weeks (n = 544) | Follow-up period  < 48 weeks (n = 184) | *p* |
| --- | --- | --- | --- | --- |
| Age, years | 75.0 [68.0–81.0] | 74.0 [68.0–80.0] | 79.0 [71.0–83.0] | <0.001^**^ |
| Sex, Female, n (%) | 422 (58.0) | 327 (60.1) | 95 (51.6) | 0.047^*^ |
| Type of vasculitis | | | | |
| MPA, n (%) | 555 (76.2) | 399 (73.3) | 156 (84.8) | 0.001^**^ |
| GPA, n (%) | 173 (23.8) | 145 (26.7) | 28 (15.2) | 0.001^**^ |
| ANCA status | | | | |
| MPO-ANCA positive, n (%) | 642 (88.2) | 475 (87.3) | 167 (90.8) | 0.236 |
| PR3-ANCA positive, n (%) | 75 (10.3) | 60 (11.0) | 15 (8.2) | 0.326 |
| negative, n (%) | 11 (1.5) | 9 (1.7) | 2 (1.1) | 0.739 |
| Comorbidity | | | | |
| Hypertension, n (%) | 332 (45.6) | 242 (44.5) | 90 (48.9) | 0.305 |
| Diabetes, n (%) | 155 (21.3) | 119 (21.9) | 36 (19.6) | 0.534 |
| Chronic kidney disease, n (%) | 114 (15.7) | 78 (14.3) | 36 (19.6) | 0.101 |
| Cardiac disease, n (%) | 109 (15.0) | 71 (13.1) | 38 (20.7) | 0.017^*^ |
| Cancer, n (%) | 104 (14.3) | 76 (14.0) | 28 (15.2) | 0.715 |
| Birmingham Vasculitis Activity Score (BVAS) | 15.0 [10.0–20.0] | 14.0 [10.0–19.0] | 16.0 [12.0–21.0] | <0.001^**^ |
| Organ involvement (BVAS ≥ 1) † | | | | |
| General, n (%) | 497 (68.3) | 373 (68.6) | 124 (67.4) | 0.784 |
| Cutaneous, n (%) | 139 (19.1) | 105 (19.3) | 34 (18.5) | 0.914 |
| Mucous membranes or eyes, n (%) | 80 (11.0) | 66 (12.1) | 14 (7.6) | 0.102 |
| Ear, nose, and throat, n (%) | 192 (26.4) | 157 (28.9) | 35 (19.0) | 0.001^**^ |
| Chest, n (%) | 334 (45.9) | 245 (45.0) | 89 (48.4) | 0.442 |
| Cardiovascular, n (%) | 35 (4.8) | 17 (3.1) | 18 (9.8) | 0.001^**^ |
| Abdominal, n (%) | 10 (1.4) | 6 (1.1) | 4 (2.2) | 0.283 |
| Renal, n (%) | 535 (73.5) | 383 (70.4) | 152 (82.6) | 0.001^**^ |
| Nervous system, n (%) | 202 (27.8) | 146 (26.8) | 56 (30.4) | 0.343 |
| Laboratory data at diagnosis | | | | |
| S-albumin, mg/dL (n = 533, n = 184) | 2.6 [2.2–3.2] | 2.6 [2.2–3.2] | 2.4 [2.0–2.9] | <0.001^**^ |
| S-creatinine, mg/dL (n = 544, n = 184) | 0.92 [0.66–1.65] | 0.87 [0.64–1.43] | 1.15 [0.70–2.30] | <0.001^**^ |
| eGFR, ml/min/1.73m^2^ (n = 544, n = 183) | 53.3 [28.1–75.4] | 55.5 [32.5–76.4] | 40.5 [19.2–70.2] | <0.001^**^ |
| Hemoglobin, mg/dL (n = 541, n = 184) | 10.3 [9.0–11.7] | 10.4 [9.2–11.8] | 9.7 [8.4–11.0] | <0.001^**^ |
| Neutrophil, /µL (n = 536, n = 179) | 8400 [5800–11950] | 8331 [5817–11920] | 8630 [5660–12290] | 0.628 |
| Lymphocyte, /µL (n = 536, n = 179) | 1201 [882–1650] | 1254 [930–1690] | 1040 [750–1500] | <0.001^**^ |
| Serum IgG, mg/dL (n = 501, n = 175) | 1628 [1314–2036] | 1627 [1323–2024] | 1638 [1277–2059] | 0.994 |
| CRP, mg/dL (n = 542, n = 184) | 8.2 [2.4–13.1] | 8.2 [2.5–13.2] | 7.9 [2.2–12.7] | 0.715 |

Data are presented as median [IQR] or as n (%), unless otherwise indicated.

ANCA, Antineutrophil Cytoplasmic Antibody; BVAS, Birmingham Vasculitis Activity Score; CRP, C-Reactive Protein; eGFR, Estimated Glomerular Filtration Rate; GFCR, Glucocorticoid-Free Clinical Remission; GPA, Granulomatosis with Polyangiitis; MPA, Microscopic Polyangiitis; MPO, Anti-Myeloperoxidase; PR3, Anti-Proteinase 3.

For statistical analyses, **p* < 0.05, ***p* < 0.01. *p*-value: Wilcoxon rank sum test, Fisher’s exact test

† Organ involvement was based on BVAS ≥ 1.
