# Supplementary material for: Predictors of glucocorticoid-free clinical remission in patients with newly diagnosed microscopic polyangiitis and granulomatosis with polyangiitis: a retrospective cohort study using a nationwide registry in Japan (J-CANVAS)
Source: Arthritis Res Ther. 2026 Mar 10;28:89. doi: 10.1186/s13075-026-03780-3 (PMC13085565; doi:10.1186/s13075-026-03780-3)
Supplement: Supplementary file 2 — Supplementary Material 2. [file 13075_2026_3780_MOESM2_ESM.docx]

Supplementary Table 2. Dosing details of rituximab and intravenous cyclophosphamide during induction and maintenance phases stratified according to GFCR status at week 48

|  | All patients (n = 544) | With GFCR (n = 29) | Without GFCR (n = 515) | *p* |
| --- | --- | --- | --- | --- |
| Induction therapy (RTX/IVCYC) (up to week 24) | | | | |
| Both RTX and IVCYC, n (%) | 19 (3.5) | 0 (0) | 19 (3.7) | 0.616 |
| RTX dose per course, mg | 585 [500–630] | - | 585 [500–630] | - |
| Number of RTX administrations | 2 [2–4] | - | 2 [2–4] | - |
| IVCYC dose per course, mg | 500 [500–670] | - | 500 [500–670] | - |
| Number of IVCYC administrations | 2 [1–4] | - | 2 [1–4] | - |
| RTX without IVCYC, n (%) | 142 (26.1) | 18 (62.1) | 124 (24.1) | <0.001^**^ |
| RTX dose per course, mg | 505 [500–600] | 565 [500–600] | 500 [500–600] | 0.446 |
| Number of RTX administrations | 4 [2–4] | 4 [4–4] | 4 [2–4] | 0.004^**^ |
| IVCYC without RTX, n (%) | 198 (36.4) | 4 (13.8) | 194 (37.7) | 0.009^**^ |
| IVCYC dose per course, mg | 500 [500–700] | 410 [319–650] | 500 [500–700] | 0.128 |
| Number of IVCYC administrations | 4 [3–6] | 5 [3–6] | 4 [3–6] | 0.591 |
| Maintenance therapy (during weeks 24–48) | | | | |
| RTX, n (%) | 71 (13.1) | 7 (24.1) | 64 (12.4) | 0.085 |
| RTX dose per course, mg | 560 [500–625] | 595 [540–600] | 556 [500–638] | 0.796 |
| Number of RTX administrations | 1 [1–2] | 2 [1–2] | 1 [1–1] | 0.017^*^ |
| Maintenance therapy (during weeks 48–96) | | | | |
| RTX, n (%) | 80 (14.7) | 11 (37.9) | 69 (13.4) | 0.001^**^ |
| RTX dose per course, mg | 545 [500–600] | 590 [500–600] | 520 [500–600] | 0.805 |
| Number of RTX administrations | 2 [1–2] | 2 [1–2] | 2 [1–2] | 0.681 |

Data are presented as median [IQR] or as n (%), unless otherwise indicated.

The typical doses of induction therapy were RTX at 375 mg/m² weekly for 4 doses and IVCYC at 15 mg/kg per dose every 2–3 weeks, in accordance with standard clinical practice in Japan, with adjustments as necessary for age, renal function, and clinical condition.

Maintenance RTX dosing and timing were determined at the discretion of the treating physician.

GFCR, Glucocorticoid-Free Clinical Remission; IVCYC, Intravenous Cyclophosphamide; RTX, Rituximab.

For statistical analyses, **p* < 0.05, ***p* < 0.01. *p*-value: Wilcoxon rank sum test, Fisher’s exact test
