# Supplementary material for: Predictors of glucocorticoid-free clinical remission in patients with newly diagnosed microscopic polyangiitis and granulomatosis with polyangiitis: a retrospective cohort study using a nationwide registry in Japan (J-CANVAS)
Source: Arthritis Res Ther. 2026 Mar 10;28:89. doi: 10.1186/s13075-026-03780-3 (PMC13085565; doi:10.1186/s13075-026-03780-3)
Supplement: Supplementary file 3 — Supplementary Material 3. [file 13075_2026_3780_MOESM3_ESM.docx]

Supplementary Table 3. Baseline characteristics (at diagnosis) of the matched cohort stratified according to GFCR status at week 48

|  | All patients (n = 109) | With GFCR (n = 28) | Without GFCR (n = 81) | *p* |
| --- | --- | --- | --- | --- |
| Age, years | 73.0 [68.5–79.0] | 74.5 [68.3–79.8] | 73.0 [68.5–78.5] | 0.827 |
| Sex, Female, n (%) | 63 (57.8) | 16 (57.1) | 47 (58.0) | 1.000 |
| Type of vasculitis | | | | |
| MPA, n (%) | 89 (81.7) | 23 (82.1) | 66 (81.5) | 1.000 |
| GPA, n (%) | 20 (18.4) | 5 (17.9) | 15 (18.5) | 1.000 |
| ANCA status | | | | |
| MPO-ANCA positive, n (%) | 101 (92.7) | 27 (96.4) | 74 (91.4) | 0.677 |
| PR3-ANCA positive, n (%) | 6 (5.5) | 1 (3.6) | 5 (6.2) | 1.000 |
| negative, n (%) | 2 (1.8) | 0 (0) | 2 (2.5) | 1.000 |
| Comorbidity | | | | |
| Hypertension, n (%) | 46 (42.2) | 14 (50.0) | 32 (39.5) | 0.379 |
| Diabetes, n (%) | 19 (17.4) | 5 (17.9) | 14 (17.3) | 1.000 |
| Chronic kidney disease, n (%) | 17 (15.6) | 4 (14.3) | 13 (16.1) | 1.000 |
| Cardiac disease, n (%) | 12 (11.0) | 2 (7.1) | 10 (12.4) | 0.727 |
| Cancer, n (%) | 6 (5.5) | 0 (0) | 6 (7.4) | 0.335 |
| Birmingham Vasculitis Activity Score (BVAS) | 15.0 [10.5–19.0] | 14.0 [8.0–20.0] | 15.0 [11.0–18.0] | 0.682 |
| Organ involvement (BVAS ≥ 1) † | | | | |
| General, n (%) | 77 (70.6) | 18 (64.3) | 59 (72.8) | 0.471 |
| Cutaneous, n (%) | 25 (22.9) | 6 (21.4) | 19 (23.5) | 1.000 |
| Mucous membranes or eyes, n (%) | 14 (12.8) | 3 (10.7) | 11 (13.6) | 1.000 |
| Ear, nose, and throat, n (%) | 30 (27.5) | 9 (32.1) | 21 (25.9) | 0.624 |
| Chest, n (%) | 51 (46.8) | 14 (50.0) | 37 (45.7) | 0.827 |
| Cardiovascular, n (%) | 2 (1.8) | 0 (0) | 2 (2.5) | 1.000 |
| Abdominal, n (%) | 0 (0) | 0 (0) | 0 (0) | - |
| Renal, n (%) | 82 (75.2) | 21 (75.0) | 61 (75.3) | 1.000 |
| Nervous system, n (%) | 29 (26.6) | 7 (25.0) | 22 (27.2) | 1.000 |
| Laboratory data at diagnosis | | | | |
| S-albumin, mg/dL (n = 27, n = 80) | 2.6 [2.2–3.1] | 2.5 [2.0–3.2] | 2.7 [2.3–3.1] | 0.500 |
| S-creatinine, mg/dL | 0.93 [0.67–1.70] | 0.87 [0.72–1.57] | 0.93 [0.64–1.79] | 0.942 |
| eGFR, ml/min/1.73m^2^ | 52.0 [28.0–74.1] | 54.5 [28.9–76.0] | 51.3 [27.8–73.9] | 0.862 |
| Hemoglobin, mg/dL (n = 28, n = 80) | 10.3 [9.0–11.6] | 10.6 [9.3–12.1] | 10.2 [8.8–10.9] | 0.071 |
| Neutrophil, /µL (n = 28, n = 80) | 8,000 [5,726–11,658] | 9,475 [4,855–11,400] | 7,835 [5,865–11,795] | 0.947 |
| Lymphocyte, /µL (n = 28, n = 80) | 1,213 [940–1,758] | 1,117 [833–1,668] | 1,233 [947–1,793] | 0.439 |
| Serum IgG, mg/dL (n = 26, n = 77) | 1,692 [1,377–2,100] | 1,659 [1,320–2,077] | 1,697 [1,382–2,130] | 0.685 |
| CRP, mg/dL | 7.4 [2.4–13.2] | 6.9 [0.5–14.1] | 7.4 [3.1–12.9] | 0.439 |

Data are presented as median [IQR] or as n (%), unless otherwise indicated.

ANCA, Antineutrophil Cytoplasmic Antibody; BVAS, Birmingham Vasculitis Activity Score; CRP, C-Reactive Protein; eGFR, Estimated Glomerular Filtration Rate; GFCR, Glucocorticoid-Free Clinical Remission; GPA, Granulomatosis with Polyangiitis; MPA, Microscopic Polyangiitis; MPO, Anti-Myeloperoxidase; PR3, Anti-Proteinase 3.

For statistical analyses, **p* < 0.05, ***p* < 0.01. *p*-value: Wilcoxon rank sum test, Fisher’s exact test

† Organ involvement was based on BVAS ≥ 1.
