# Supplementary material for: Predictors of glucocorticoid-free clinical remission in patients with newly diagnosed microscopic polyangiitis and granulomatosis with polyangiitis: a retrospective cohort study using a nationwide registry in Japan (J-CANVAS)
Source: Arthritis Res Ther. 2026 Mar 10;28:89. doi: 10.1186/s13075-026-03780-3 (PMC13085565; doi:10.1186/s13075-026-03780-3)
Supplement: Supplementary file 4 — Supplementary Material 4. [file 13075_2026_3780_MOESM4_ESM.docx]

Supplementary Table 4. Treatment details and outcomes (up to week 48) of the matched cohort stratified according to GFCR status at week 48

|  | All patients (n = 109) | With GFCR (n = 28) | Without GFCR (n = 81) | *p* |
| --- | --- | --- | --- | --- |
| Year in which remission induction therapy was initiated, n (%) | | | | |
| 2017–2018 | 43 (39.5) | 12 (42.9) | 31 (38.3) | 0.662 |
| 2019–2020 | 33 (30.3) | 6 (21.4) | 27 (33.3) | 0.340 |
| 2021–2023 | 33 (30.3) | 10 (35.7) | 23 (28.4) | 0.482 |
| Daily GC dose (prednisolone-equivalent) at each time point | | | | |
| Initial dose, mg | 45.0 [30.0–50.0] | 32.5 [30.0–50.0] | 50.0 [36.5–55.0] | 0.007^**^ |
| at week 1, mg | 40.0 [30.0–50.0] | 30.0 [26.3–50.0] | 40.0 [35.0–50.0] | 0.020^*^ |
| at week 2, mg | 37.5 [25.0–50.0] | 27.5 [15.0–40.0] | 40.0 [30.0–50.0] | <0.001^**^ |
| at week 4, mg | 30.0 [20.0–35.0] | 20.0 [7.5–30.0] | 30.0 [25.0–38.8] | <0.001^**^ |
| at week 8, mg | 20.0 [14.0–25.0] | 13.8 [4.0–20.0] | 20.0 [15.5–25.0] | <0.001^**^ |
| at week 12, mg | 15.0 [10.0–20.0] | 6.3 [2.0–14.4] | 17.5 [12.5–20.0] | <0.001^**^ |
| at week 16, mg | 12.5 [7.8–16.5] | 3.0 [0–10.0] | 15.0 [10.0–17.5] | <0.001^**^ |
| at week 20, mg | 10.0 [6.0–15.0] | 1.3 [0–7.4] | 12.5 [10.0–15.0] | <0.001^**^ |
| at week 24, mg | 10.0 [5.0–13.0] | 0.5 [0–5.0] | 10.0 [7.5–15.0] | <0.001^**^ |
| at week 48, mg | 5.0 [0–9.5] | 0 [0–0] | 6.0 [5.0–10.0] | <0.001^**^ |
| Treatment up to week 24 | | | | |
| Induction therapy (RTX/IVCYC) | | | | |
| Both RTX and IVCYC, n (%) | 0 (0) | 0 (0) | 0 (0) | - |
| RTX without IVCYC, n (%) | 47 (43.1) | 17 (60.7) | 30 (37.0) | 0.045^*^ |
| IVCYC without RTX, n (%) | 29 (26.6) | 4 (14.3) | 25 (30.9) | 0.135 |
| Neither RTX nor IVCYC, n (%) | 33 (30.3) | 7 (25.0) | 26 (32.1) | 0.634 |
| Other immunosuppressive agents | | | | |
| AZA, n (%) | 30 (27.5) | 5 (17.9) | 25 (30.9) | 0.226 |
| MMF, n (%) | 2 (1.8) | 0 (0) | 2 (2.5) | 1.000 |
| MTX, n (%) | 4 (3.7) | 1 (3.6) | 3 (3.7) | 1.000 |
| MZR, n (%) | 5 (4.6) | 2 (7.1) | 3 (3.7) | 0.601 |
| Adjunctive therapy | | | | |
| Methylprednisolone pulse, n (%) | 29 (26.7) | 2 (7.1) | 27 (33.3) | 0.006^**^ |
| PLEX, n (%) | 4 (3.7) | 0 (0) | 4 (4.9) | 0.571 |
| Avacopan, n (%) | 7 (6.4) | 6 (21.4) | 1 (1.2) | 0.001^**^ |
| Treatment from weeks 24–48 | | | | |
| Maintenance therapy | | | | |
| RTX, n (%) | 20 (18.4) | 7 (25.0) | 13 (16.1) | 0.395 |
| AZA, n (%) | 36 (33.0) | 7 (25.0) | 29 (35.8) | 0.356 |
| MMF, n (%) | 4 (3.7) | 1 (3.6) | 3 (3.7) | 1.000 |
| MTX, n (%) | 6 (5.5) | 1 (3.6) | 5 (6.2) | 1.000 |
| MZR, n (%) | 8 (7.3) | 1 (3.6) | 7 (8.6) | 0.677 |
| Adjunctive therapy | | | | |
| Avacopan, n (%) | 5 (4.6) | 4 (14.3) | 1 (1.2) | 0.015^*^ |
| Outcomes up to week 48 | | | | |
| Death, n (%) | 0 (0) | 0 (0) | 0 (0) | - |
| Major relapse, n (%) | 0 (0) | 0 (0) | 0 (0) | - |
| Minor relapse, n (%) | 6 (5.5) | 0 (0) | 6 (7.4) | 0.335 |
| Severe infection, n (%) | 6 (5.5) | 0 (0) | 6 (7.4) | 0.335 |
| Renal outcomes through week 48 among patients with renal involvement at baseline (all patients, n = 82; with GFCR, n = 21; without GFCR, n = 61) † | | | | |
| eGFR at baseline, ml/min/1.73 m^2^ (n = 21, n = 61) | 39.6 [23.6–57.8] | 38.7 [23.5–66.7] | 40.6 [23.5–56.9] | 0.746 |
| eGFR at week 4, ml/min/1.73 m^2^ (n = 21, n = 59) | 39.7 [28.5–57.8] | 39.3 [28.9–65.9] | 39.7 [28.4–57.3] | 0.607 |
| eGFR at week 12, ml/min/1.73 m^2^ (n = 21, n = 61) | 41.6 [31.6–55.8] | 44.1 [32.5–60.8] | 41.1 [30.1–55.9] | 0.500 |
| eGFR at week 24, ml/min/1.73 m^2^ (n = 21, n = 60) | 41.0 [31.5–56.0] | 41.5 [31.5–60.9] | 39.8 [31.4–55.6] | 0.647 |
| eGFR at week 48, ml/min/1.73 m^2^ (n = 21, n = 60) | 39.7 [32.8–54.8] | 46.2 [36.0–56.3] | 39.1 [32.2–53.9] | 0.242 |
| Changes in eGFR from baseline to week 48, ml/min/1.73 m² (n = 21, n = 60) | 0.2 [−7.2–8.9] | 0.7 [−6.5–12.3] | −0.6 [−8.8–8.5] | 0.368 |
| Progression to end-stage kidney disease, n (%) ‡ | 3 (3.7) | 1 (4.8) | 2 (3.3) | 1.000 |

Data are presented as median [IQR] or as n (%), unless otherwise indicated.

AZA, Azathioprine; eGFR, Estimated Glomerular Filtration Rate; GC, Glucocorticoid; GFCR, Glucocorticoid-Free Clinical Remission; IVCYC, Intravenous Cyclophosphamide; MMF, Mycophenolate Mofetil; MTX, methotrexate; MZR, Mizoribine; PLEX, Plasma Exchange; RTX, Rituximab.

For statistical analyses, **p* < 0.05, ***p* < 0.01. *p*-value: Wilcoxon rank sum test, Fisher’s exact test

† Renal involvement was defined as BVAS renal item ≥ 1.

‡ End-stage kidney disease was defined as an eGFR < 15 mL/min/1.73 m² or permanent kidney replacement therapy (hemodialysis or peritoneal dialysis).
