# Supplementary material for: Predictors of glucocorticoid-free clinical remission in patients with newly diagnosed microscopic polyangiitis and granulomatosis with polyangiitis: a retrospective cohort study using a nationwide registry in Japan (J-CANVAS)
Source: Arthritis Res Ther. 2026 Mar 10;28:89. doi: 10.1186/s13075-026-03780-3 (PMC13085565; doi:10.1186/s13075-026-03780-3)
Supplement: Supplementary file 5 — Supplementary Material 5. [file 13075_2026_3780_MOESM5_ESM.docx]

Supplementary Table 5. Univariable and multivariable logistic regression analyses for predicting GFCR at week 48 in the matched cohort

| Factors | Univariable analysis | | Multivariable analysis, model 1† | | Multivariable analysis, model 2‡ | |
| --- | --- | --- | --- | --- | --- | --- |
|  | OR (95% CI) | *p* | OR (95% CI) | *p* | OR (95% CI) | *p* |
| Age | 1.00 (0.94–1.05) | 0.888 |  |  |  |  |
| Female | 0.96 (0.40–2.30) | 0.935 |  |  |  |  |
| MPA | 1.05 (0.34–3.20) | 0.938 |  |  |  |  |
| MPO-ANCA positive | 2.55 (0.30–21.73) | 0.391 |  |  |  |  |
| BVAS | 0.99 (0.93–1.05) | 0.714 |  |  |  |  |
| Organ involvement (BVAS ≥ 1) † | | | | | | |
| General | 0.67 (0.27–1.68) | 0.393 |  |  |  |  |
| Cutaneous | 0.89 (0.31–2.51) | 0.826 |  |  |  |  |
| Mucous Membranes or eyes | 0.76 (0.20–2.96) | 0.697 |  |  |  |  |
| Ear, nose, and throat | 1.35 (0.53–3.45) | 0.526 |  |  |  |  |
| Chest | 1.19 (0.50–2.81) | 0.693 |  |  |  |  |
| Renal | 0.98 (0.36–2.66) | 0.974 |  |  |  |  |
| Nervous system | 0.89 (0.33–2.40) | 0.824 |  |  |  |  |
| Laboratory data at diagnosis | | | | | | |
| S-albumin, mg/dL | 0.87 (0.44–1.69) | 0.675 |  |  |  |  |
| eGFR, ml/min/1.73m^2^ | 1.00 (0.99–1.02) | 0.971 |  |  |  |  |
| CRP, mg/dL | 0.98 (0.91–1.05) | 0.583 |  |  |  |  |
| Induction therapy up to 24 weeks | | | | | | |
| RTX without IVCYC | 2.63 (1.09–6.35) | 0.032^*^ | 3.04 (1.12–8.26) | 0.029^*^ | 2.37 (0.77–7.31) | 0.133 |
| IVCYC without RTX | 0.37 (0.12–1.19) | 0.096 |  |  | 0.51 (0.11–2.47) | 0.407 |
| Neither RTX nor IVCYC | 0.71 (0.27–1.87) | 0.482 |  |  |  |  |
| Maintenance therapy from 24 to 48 weeks | | | | | | |
| RTX | 1.74 (0.62–4.94) | 0.295 |  |  |  |  |
| AZA | 0.60 (0.23–1.57) | 0.298 |  |  |  |  |
| Adjunctive therapy | | | | | | |
| Use of methylprednisolone pulse up to 24 weeks | 0.15 (0.03–0.70) | 0.015^*^ | 0.15 (0.03–0.75) | 0.021^*^ | 0.14 (0.03–0.76) | 0.023^*^ |
| Use of avacopan up to 48 weeks | 21.8 (2.5–190.9) | 0.005^**^ | 32.3 (2.8–378.2) | 0.006^**^ | 33.3 (3.0–370.5) | 0.004^**^ |

Logistic regression models were applied for univariate and multivariate analysis.

†p-values are from model 1 of multivariable logistic regression including factors with p-values < 0.05 in univariable analysis.

‡p-values are from model 2 of multivariable logistic regression including factors with p-values < 0.20 in univariable analysis.

ANCA, Antineutrophil Cytoplasmic Antibody; AZA, Azathioprine; BVAS, Birmingham Vasculitis Activity Score; CRP, C-Reactive Protein; eGFR, Estimated Glomerular Filtration Rate; IVCYC, Intravenous Cyclophosphamide; MPA, Microscopic Polyangiitis; MPO, Anti-Myeloperoxidase; RTX, Rituximab.

For statistical analyses, **p* < 0.05, ***p* < 0.01.
