# Supplementary material for: Predictors of glucocorticoid-free clinical remission in patients with newly diagnosed microscopic polyangiitis and granulomatosis with polyangiitis: a retrospective cohort study using a nationwide registry in Japan (J-CANVAS)
Source: Arthritis Res Ther. 2026 Mar 10;28:89. doi: 10.1186/s13075-026-03780-3 (PMC13085565; doi:10.1186/s13075-026-03780-3)
Supplement: Supplementary file 6 — Supplementary Material 6. [file 13075_2026_3780_MOESM6_ESM.docx]

Supplementary Table 6. Baseline characteristics (at diagnosis) of patients treated with rituximab versus cyclophosphamide in the restricted cohort

|  | All patients (n = 202) | RTX without IVCYC (n = 95) | IVCYC without RTX (n = 107) | *p* |
| --- | --- | --- | --- | --- |
| Age, years | 75.0 [68.0–79.0] | 76.0 [68.0–81.0] | 74.0 [68.0–77.0] | 0.165 |
| Sex, Female, n (%) | 121 (59.9) | 57 (60.0) | 64 (59.8) | 1.000 |
| Type of vasculitis | | | | |
| MPA, n (%) | 140 (69.3) | 67 (70.5) | 73 (68.2) | 0.761 |
| GPA, n (%) | 62 (30.7) | 28 (29.5) | 34 (31.8) | 0.761 |
| ANCA status | | | | |
| MPO-ANCA positive, n (%) | 178 (88.1) | 82 (86.3) | 96 (89.7) | 0.517 |
| PR3-ANCA positive, n (%) | 23 (11.4) | 13 (13.7) | 10 (9.4) | 0.379 |
| negative, n (%) | 1 (0.5) | 0 (0) | 1 (0.9) | 1.000 |
| Comorbidity | | | | |
| Hypertension, n (%) | 100 (49.5) | 45 (47.4) | 55 (51.4) | 0.576 |
| Diabetes, n (%) | 47 (23.3) | 15 (15.8) | 32 (29.9) | 0.020^*^ |
| Chronic kidney disease, n (%) | 19 (9.4) | 13 (13.7) | 6 (5.6) | 0.057 |
| Cardiac disease, n (%) | 19 (9.4) | 12 (12.6) | 7 (6.5) | 0.155 |
| Cancer, n (%) | 15 (7.4) | 7 (7.4) | 8 (7.5) | 1.000 |
| Birmingham Vasculitis Activity Score (BVAS) | 14.0 [10.0–19.0] | 13.0 [9.0–17.0] | 14.0 [10.0–19.0] | 0.435 |
| Organ involvement (BVAS ≥ 1) † | | | | |
| General, n (%) | 140 (69.3) | 64 (67.4) | 76 (71.0) | 0.647 |
| Cutaneous, n (%) | 44 (21.8) | 25 (26.3) | 19 (17.8) | 0.172 |
| Mucous membranes or eyes, n (%) | 28 (13.9) | 13 (13.7) | 15 (14.0) | 1.000 |
| Ear, nose, and throat, n (%) | 67 (33.2) | 30 (31.6) | 37 (34.6) | 0.657 |
| Chest, n (%) | 87 (43.1) | 37 (39.0) | 50 (46.7) | 0.319 |
| Cardiovascular, n (%) | 4 (2.0) | 1 (1.1) | 3 (2.8) | 0.624 |
| Abdominal, n (%) | 2 (1.0) | 2 (2.1) | 0 (0) | 0.220 |
| Renal, n (%) | 146 (72.3) | 71 (74.7) | 75 (70.1) | 0.530 |
| Nervous system, n (%) | 52 (25.7) | 20 (21.1) | 32 (29.9) | 0.197 |
| Laboratory data at diagnosis | | | | |
| S-albumin, mg/dL (n = 94, n = 105) | 2.7 [2.3–3.3] | 2.8 [2.4–3.2] | 2.6 [2.3–3.3] | 0.845 |
| S-creatinine, mg/dL | 0.82 [0.64–1.17] | 0.83 [0.65–1.19] | 0.80 [0.64–1.16] | 0.501 |
| eGFR, ml/min/1.73 m^2^ | 58.4 [41.1–77.9] | 55.5 [41.1–77.1] | 62.3 [40.9–78.9] | 0.554 |
| Hemoglobin, mg/dL (n = 95, n = 106) | 10.4 [9.2–12.0] | 10.2 [9.2–12.0] | 10.5 [9.2–12.0] | 0.722 |
| Neutrophil, /µL (n = 94, n = 106) | 7,968 [5,487–11,363] | 7,490 [5,075–11,037] | 8,220 [5,496–11,761] | 0.419 |
| Lymphocyte, /µL (n = 94, n = 106) | 1,246 [967–1,697] | 1,250 [920–1,617] | 1,246 [1,005–1,770] | 0.416 |
| Serum IgG, mg/dL (n = 88, n = 98) | 1,636 [1,304–2,012] | 1,666 [1,301–2,021] | 1,588 [1,304–1,997] | 0.717 |
| CRP, mg/dL | 8.1 [2.2–13.0] | 7.8 [2.0–13.7] | 8.1 [2.3–12.5] | 0.656 |

The restricted cohort excluded patients who received both RTX and IVCYC, neither RTX nor IVCYC, methylprednisolone pulse therapy, plasma exchange, or avacopan.

Data are presented as median [IQR] or as n (%), unless otherwise indicated.

ANCA, Antineutrophil Cytoplasmic Antibody; BVAS, Birmingham Vasculitis Activity Score; CRP, C-Reactive Protein; eGFR, Estimated Glomerular Filtration Rate; GPA, Granulomatosis with Polyangiitis; IVCYC, Intravenous Cyclophosphamide; MPA, Microscopic Polyangiitis; MPO, Anti-Myeloperoxidase; PR3, Anti-Proteinase 3; RTX, Rituximab.

For statistical analyses, **p* < 0.05, ***p* < 0.01. *p*-value: Wilcoxon rank sum test, Fisher’s exact test

† Organ involvement was based on BVAS ≥ 1.
