# Supplementary material for: Predictors of glucocorticoid-free clinical remission in patients with newly diagnosed microscopic polyangiitis and granulomatosis with polyangiitis: a retrospective cohort study using a nationwide registry in Japan (J-CANVAS)
Source: Arthritis Res Ther. 2026 Mar 10;28:89. doi: 10.1186/s13075-026-03780-3 (PMC13085565; doi:10.1186/s13075-026-03780-3)
Supplement: Supplementary file 7 — Supplementary Material 7. [file 13075_2026_3780_MOESM7_ESM.docx]

Supplementary Table 7. Treatment details and outcomes (up to week 48) of patients treated with rituximab versus intravenous cyclophosphamide in the restricted cohort

|  | All patients (n = 202) | RTX without IVCYC (n = 95) | IVCYC without RTX (n = 107) | *p* |
| --- | --- | --- | --- | --- |
| Year in which remission induction therapy was initiated, n (%) | | | | |
| 2017–2018 | 73 (36.1) | 27 (28.4) | 46 (43.0) | 0.040^*^ |
| 2019–2020 | 67 (33.2) | 30 (31.6) | 37 (34.6) | 0.657 |
| 2021–2023 | 62 (30.7) | 38 (40.0) | 24 (22.4) | 0.009^**^ |
| Daily GC dose (prednisolone-equivalent) at each time point | | | | |
| Initial dose, mg | 45.0 [35.0–55.0] | 45.0 [30.0–55.0] | 45.0 [38.0–55.0] | 0.881 |
| Initial dose, mg/kg (n = 94, n = 107) | 0.86 [0.69–1.00] | 0.86 [0.63–1.04] | 0.85 [0.73–0.98] | 0.842 |
| at week 1, mg | 45.0 [34.8–50.0] | 40.0 [30.0–50.0] | 45.0 [38.0–50.0] | 0.177 |
| at week 2, mg | 40.0 [30.0–45.3] | 35.0 [25.0–40.0] | 40.0 [30.0–50.0] | <0.001^**^ |
| at week 4, mg | 30.0 [20.0–40.0] | 25.0 [20.0–30.0] | 35.0 [30.0–45.0] | <0.001^**^ |
| at week 8, mg | 20.0 [15.0–25.5] | 17.5 [12.5–20.0] | 25.0 [20.0–30.0] | <0.001^**^ |
| at week 12, mg | 15.0 [12.5–20.0] | 12.5 [10.0–16.0] | 19.0 [15.0–21.0] | <0.001^**^ |
| at week 16, mg | 13.0 [10.0–17.5] | 10.0 [7.5–15.0] | 15.0 [12.5–18.0] | <0.001^**^ |
| at week 20, mg | 10.8 [8.0–15.0] | 10.0 [6.0–12.0] | 13.0 [10.0–15.0] | <0.001^**^ |
| at week 24, mg | 10.0 [7.0–12.5] | 8.0 [5.0–10.0] | 11.0 [10.0–14.0] | <0.001^**^ |
| at week 48, mg | 6.0 [4.0–9.0] | 5.0 [3.0–6.0] | 7.5 [5.0–10.0] | <0.001^**^ |
| Treatment up to week 24 | | | | |
| Immunosuppressive agents other than RTX and IVCYC | | | | |
| AZA, n (%) | 76 (37.6) | 26 (27.4) | 50 (46.7) | 0.006^**^ |
| MMF, n (%) | 1 (0.5) | 0 (0) | 1 (0.9) | 1.000 |
| MTX, n (%) | 5 (2.5) | 2 (2.1) | 3 (2.8) | 1.000 |
| MZR, n (%) | 9 (4.5) | 9 (9.5) | 0 (0) | <0.001^**^ |
| Treatment from week 24–48 | | | | |
| Maintenance therapy | | | | |
| RTX, n (%) | 39 (19.3) | 34 (35.8) | 5 (4.7) | <0.001^**^ |
| AZA, n (%) | 76 (37.6) | 26 (27.4) | 50 (46.7) | 0.006^**^ |
| MMF, n (%) | 1 (0.5) | 0 (0) | 1 (0.9) | 1.000 |
| MTX, n (%) | 5 (2.5) | 2 (2.1) | 3 (2.8) | 1.000 |
| MZR, n (%) | 9 (4.5) | 9 (9.5) | 0 (0) | <0.001^**^ |
| Outcomes up to week 48 | | | | |
| GFCR at week 48 | 16 (7.9) | 14 (14.7) | 2 (1.9) | 0.001^**^ |
| Death, n (%) | 0 (0) | 0 (0) | 0 (0) | - |
| Major relapse, n (%) | 0 (0) | 0 (0) | 0 (0) | - |
| Minor relapse, n (%) | 8 (4.0) | 3 (3.2) | 5 (4.7) | 0.725 |
| Severe infection, n (%) | 11 (5.5) | 8 (8.4) | 3 (2.8) | 0.119 |

The restricted cohort excluded patients who received both RTX and IVCYC, neither RTX nor IVCYC, methylprednisolone pulse therapy, plasma exchange, or avacopan.

Data are presented as median [IQR] or as n (%), unless otherwise indicated.

AZA, Azathioprine; GC, Glucocorticoid; GFCR, Glucocorticoid-Free Clinical Remission; IVCYC, Intravenous Cyclophosphamide; MMF, Mycophenolate Mofetil; MTX, Methotrexate; MZR, Mizoribine; RTX, Rituximab.

For statistical analyses, **p* < 0.05, ***p* < 0.01. *P*-value: Wilcoxon rank sum test, Fisher’s exact test
