# Supplementary material for: Predictors of glucocorticoid-free clinical remission in patients with newly diagnosed microscopic polyangiitis and granulomatosis with polyangiitis: a retrospective cohort study using a nationwide registry in Japan (J-CANVAS)
Source: Arthritis Res Ther. 2026 Mar 10;28:89. doi: 10.1186/s13075-026-03780-3 (PMC13085565; doi:10.1186/s13075-026-03780-3)
Supplement: Supplementary file 8 — Supplementary Material 8. [file 13075_2026_3780_MOESM8_ESM.docx]

Supplementary Table 8. Long-term clinical outcomes (weeks 48–96) in the matched cohort stratified according to GFCR status at week 48

|  | All patients (n = 109) | With GFCR (n = 28) | Without GFCR (n = 81) | *p* |
| --- | --- | --- | --- | --- |
| Discontinuation of follow-up between weeks 48 and 96 | 19 (17.4) | 9 (32.1) | 10 (12.4) | 0.039^*^ |
| Death, n (%) | 1 (0.9) | 1 (3.6) | 0 (0) | 0.257 |
| of which, due to vasculitis-worsening or infections, n (%) | 0 (0) | 0 (0) | 0 (0) | - |
| Major relapse, n (%) | 0 (0) | 0 (0) | 0 (0) | - |
| Lost to follow-up due to transfer, n (%) | 5 (4.6) | 3 (10.7) | 2 (2.5) | 0.106 |
| Study period ended before completion of the 96-week follow-up, n (%) | 13 (11.9) | 5 (17.9) | 8 (9.9) | 0.312 |
| Completed 96-week follow-up | 90 (82.6) | 19 (67.9) | 71 (87.7) | 0.039^*^ |
| GFCR at week 96 | 20 (18.4) | 16 (57.1) | 4 (4.9) | <0.001^**^ |
| Daily GC dose at week 96, mg (N = 19, N = 71) | 5.0 [1.0–5.0] | 0 [0–0] | 5.0 [4.0–6.0] | <0.001^**^ |
| Minor relapse between weeks 48 and 96, n (%) | 7 (6.4) | 1 (3.6) | 6 (7.4) | 0.675 |
| Serious infection between weeks 48 and 96, n (%) | 0 (0) | 0 (0) | 0 (0) | - |
| Treatment between weeks 48 and 96 | | | | |
| RTX (maintenance), n (%) | 26 (23.9) | 10 (35.7) | 16 (19.8) | 0.122 |
| AZA, n (%) | 33 (30.3) | 7 (25.0) | 26 (32.1) | 0.634 |
| MMF, n (%) | 4 (3.7) | 1 (3.6) | 3 (3.7) | 1.000 |
| MTX, n (%) | 5 (4.6) | 1 (3.6) | 4 (4.9) | 1.000 |
| MZR, n (%) | 8 (7.3) | 1 (3.6) | 7 (8.6) | 0.677 |
| Avacopan, n (%) | 7 (6.4) | 4 (14.3) | 3 (3.7) | 0.070 |

Data are presented as median [IQR] or as n (%), unless otherwise indicated.

AZA, Azathioprine; GC, Glucocorticoid; GFCR, Glucocorticoid-Free Clinical Remission; MMF, Mycophenolate Mofetil; MTX, Methotrexate; MZR, Mizoribine; RTX, Rituximab.

For statistical analyses, **p* < 0.05, ***p* < 0.01. *p*-value: Wilcoxon rank sum test, Fisher’s exact test
