# Supplementary material for: Predictors of glucocorticoid-free clinical remission in patients with newly diagnosed microscopic polyangiitis and granulomatosis with polyangiitis: a retrospective cohort study using a nationwide registry in Japan (J-CANVAS)
Source: Arthritis Res Ther. 2026 Mar 10;28:89. doi: 10.1186/s13075-026-03780-3 (PMC13085565; doi:10.1186/s13075-026-03780-3)
Supplement: Supplementary file 9 — Supplementary Material 9. [file 13075_2026_3780_MOESM9_ESM.docx]

Supplementary Table 9. Baseline characteristics (at diagnosis) of patients stratified according to methylprednisolone pulse therapy use

|  | All patients (n = 544) | With methylprednisolone pulse (n = 177) | Without methylprednisolone pulse (n = 367) | *p* |
| --- | --- | --- | --- | --- |
| Age, years | 74.0 [68.0–80.0] | 73.0 [67.0–79.5] | 75.0 [68.0–80.0] | 0.236 |
| Sex, Female, n (%) | 327 (60.1) | 91 (51.4) | 236 (64.3) | 0.005^**^ |
| Type of vasculitis | | | | |
| MPA, n (%) | 399 (73.3) | 134 (75.7) | 265 (72.2) | 0.409 |
| GPA, n (%) | 145 (26.7) | 43 (24.3) | 102 (27.8) | 0.409 |
| ANCA status | | | | |
| MPO-ANCA positive, n (%) | 475 (87.3) | 154 (87.0) | 321 (87.5) | 0.891 |
| PR3-ANCA positive, n (%) | 60 (11.0) | 20 (11.3) | 40 (10.9) | 0.885 |
| negative, n (%) | 9 (1.7) | 3 (1.7) | 6 (1.6) | 1.000 |
| Comorbidity | | | | |
| Hypertension, n (%) | 242 (44.5) | 82 (46.3) | 160 (43.6) | 0.581 |
| Diabetes, n (%) | 119 (21.9) | 39 (22.0) | 80 (21.8) | 1.000 |
| Chronic kidney disease, n (%) | 78 (14.3) | 32 (18.1) | 46 (12.5) | 0.091 |
| Cardiac disease, n (%) | 71 (13.1) | 26 (14.7) | 45 (12.3) | 0.419 |
| Cancer, n (%) | 37 (6.8) | 15 (8.5) | 22 (6.0) | 0.281 |
| Birmingham Vasculitis Activity Score (BVAS) | 14.0 [10.0–19.0] | 16.0 [13.0–21.0] | 13.0 [8.0–18.0] | <0.001^**^ |
| Organ involvement (BVAS ≥ 1) † | | | | |
| General, n (%) | 373 (68.6) | 118 (66.7) | 255 (69.5) | 0.554 |
| Cutaneous, n (%) | 105 (19.3) | 32 (18.1) | 73 (19.9) | 0.645 |
| Mucous membranes or eyes, n (%) | 66 (12.1) | 22 (12.4) | 44 (12.0) | 0.889 |
| Ear, nose, and throat, n (%) | 157 (28.9) | 44 (24.9) | 113 (30.8) | 0.159 |
| Chest, n (%) | 245 (45.0) | 94 (53.1) | 151 (41.1) | 0.010^*^ |
| Cardiovascular, n (%) | 17 (3.1) | 8 (4.5) | 9 (2.5) | 0.199 |
| Abdominal, n (%) | 6 (1.1) | 1 (0.6) | 5 (1.4) | 0.669 |
| Renal, n (%) | 383 (70.4) | 146 (82.5) | 237 (64.6) | <0.001^**^ |
| Nervous system, n (%) | 146 (26.8) | 47 (26.6) | 99 (27.0) | 1.000 |
| Laboratory data at diagnosis | | | | |
| S-albumin, mg/dL (n = 174, n = 359) | 2.6 [2.2–3.2] | 2.5 [2.1–3.2] | 2.7 [2.3–3.2] | 0.042^*^ |
| S-creatinine, mg/dL | 0.87 [0.64–1.43] | 1.26 [0.77–2.19] | 0.78 [0.62–1.14] | <0.001^**^ |
| eGFR, ml/min/1.73 m^2^ | 55.5 [32.5–76.4] | 41.1 [20.3–64.5] | 62.4 [41.4–79.3] | <0.001^**^ |
| Hemoglobin, mg/dL (n = 176, n = 365) | 10.4 [9.2–11.8] | 10.4 [9.1–11.6] | 10.4 [9.3–11.9] | 0.205 |
| Neutrophil, /µL (n = 172, n = 364) | 8,331 [5,817–11,920] | 9,139 [6,197–13,518] | 7,926 [5,500–11,980] | <0.001^**^ |
| Lymphocyte, /µL (n = 172, n = 364) | 1254 [930–1690] | 1,184 [855–1,635] | 1,288 [979–1,708] | 0.014^*^ |
| Serum IgG, mg/dL (n = 171, n = 330) | 1627 [1323–2024] | 1,570 [1,316–1,875] | 1,669 [1,324–2,044] | 0.149 |
| CRP, mg/dL (n = 176, n = 366) | 8.2 [2.5–13.2] | 8.6 [3.6–14.6] | 8.1 [2.2–12.6] | 0.029^*^ |

Data are presented as median [IQR] or as n (%), unless otherwise indicated.

ANCA, Antineutrophil Cytoplasmic Antibody; BVAS, Birmingham Vasculitis Activity Score; CRP, C-Reactive Protein; eGFR, Estimated Glomerular Filtration Rate; GFCR, Glucocorticoid-Free Clinical Remission; GPA, Granulomatosis with Polyangiitis; MPA, Microscopic Polyangiitis; MPO, Anti-Myeloperoxidase; PR3, Anti-Proteinase 3.

For statistical analyses, **p* < 0.05, ***p* < 0.01. *p*-value: Wilcoxon rank sum test, Fisher’s exact test

† Organ involvement was based on BVAS ≥ 1.
