# Supplementary material for: Predictors of glucocorticoid-free clinical remission in patients with newly diagnosed microscopic polyangiitis and granulomatosis with polyangiitis: a retrospective cohort study using a nationwide registry in Japan (J-CANVAS)
Source: Arthritis Res Ther. 2026 Mar 10;28:89. doi: 10.1186/s13075-026-03780-3 (PMC13085565; doi:10.1186/s13075-026-03780-3)
Supplement: Supplementary file 10 — Supplementary Material 10. [file 13075_2026_3780_MOESM10_ESM.docx]

Supplementary Table 10. Treatment details and outcomes (up to week 48) of patients stratified according to methylprednisolone pulse therapy use

|  | All patients (n = 544) | With methylprednisolone pulse (n = 177) | Without methylprednisolone pulse (n = 367) | *p* |
| --- | --- | --- | --- | --- |
| Year in which remission induction therapy was initiated, n (%) | | | | |
| 2017–2018 | 196 (36.0) | 57 (32.2) | 139 (37.9) | 0.216 |
| 2019–2020 | 182 (33.5) | 65 (36.7) | 117 (31.9) | 0.286 |
| 2021–2023 | 166 (30.5) | 55 (31.1) | 111 (30.3) | 0.843 |
| Daily GC dose (prednisolone-equivalent) at each time point | | | | |
| Initial dose, mg | 40.0 [30.0–50.0] | 40.0 [37.5–50.0] | 40.0 [30.0–50.0] | 0.142 |
| Initial dose, mg/kg (n = 177, n = 364) | 0.82 [0.64–0.98] | 0.83 [0.68–1.00] | 0.81 [0.63–0.98] | 0.245 |
| at week 1, mg | 40.0 [30.0–50.0] | 40.0 [35.0–51.5] | 40.0 [30.0–50.0] | 0.006^**^ |
| at week 2, mg | 40.0 [30.0–45.0] | 40.0 [30.0–50.0] | 35.0 [30.0–45.0] | <0.001^**^ |
| at week 4, mg | 30.0 [25.0–40.0] | 30.0 [25.5–40.0] | 30.0 [20.0–35.0] | <0.001^**^ |
| at week 8, mg | 20.0 [17.0–27.0] | 25.0 [20.0–30.0] | 20.0 [15.0–25.0] | <0.001^**^ |
| at week 12, mg | 17.5 [12.5–20.0] | 18.0 [15.0–21.0] | 15.0 [12.5–20.0] | <0.001^**^ |
| at week 16, mg | 15.0 [10.0–17.5] | 15.0 [12.5–20.0] | 15.0 [10.0–17.5] | <0.001^**^ |
| at week 20, mg | 12.5 [9.0–15.0] | 13.0 [10.0–16.0] | 12.5 [9.0–15.0] | 0.002^**^ |
| at week 24, mg | 10.0 [8.0–14.0] | 12.0 [9.0–15.0] | 10.0 [7.5–13.5] | 0.005^**^ |
| at week 48, mg | 7.0 [5.0–9.0] | 7.0 [5.0–10.0] | 6.0 [5.0–9.0] | 0.035^*^ |
| Treatment up to week 24 | | | | |
| Induction therapy (RTX/IVCYC) | | | | |
| Both RTX and IVCYC, n (%) | 19 (3.5) | 9 (5.1) | 10 (2.7) | 0.211 |
| RTX without IVCYC, n (%) | 142 (26.1) | 42 (23.7) | 100 (27.3) | 0.406 |
| IVCYC without RTX, n (%) | 198 (36.4) | 88 (49.7) | 110 (30.0) | <0.001^**^ |
| Neither RTX nor IVCYC, n (%) | 185 (34.0) | 38 (21.5) | 147 (40.1) | <0.001^**^ |
| Other immunosuppressive agents | | | | |
| AZA, n (%) | 182 (33.5) | 40 (22.6) | 142 (38.7) | <0.001^**^ |
| MMF, n (%) | 11 (2.0) | 4 (2.3) | 7 (1.9) | 0.7542 |
| MTX, n (%) | 20 (3.7) | 1 (0.6) | 19 (5.2) | 0.006^**^ |
| MZR, n (%) | 20 (3.7) | 1 (0.6) | 19 (5.2) | 0.006^**^ |
| Adjunctive therapy | | | | |
| Methylprednisolone pulse, n (%) | 177 (32.5) | 177 (100.0) | 0 (0) | <0.001^**^ |
| PLEX, n (%) | 29 (5.3) | 25 (14.1) | 4 (1.1) | <0.001^**^ |
| Avacopan, n (%) | 15 (2.8) | 7 (4.0) | 8 (2.2) | 0.267 |
| Treatment from weeks 24–48 | | | | |
| Maintenance therapy | | | | |
| RTX, n (%) | 71 (13.1) | 27 (15.3) | 44 (12.0) | 0.342 |
| AZA, n (%) | 222 (40.8) | 68 (38.4) | 154 (42.0) | 0.457 |
| MMF, n (%) | 24 (4.4) | 6 (3.4) | 18 (4.9) | 0.509 |
| MTX, n (%) | 29 (5.3) | 5 (2.8) | 24 (6.5) | 0.101 |
| MZR, n (%) | 30 (5.5) | 3 (1.7) | 27 (7.4) | 0.005^**^ |
| Adjunctive therapy | | | | |
| Avacopan, n (%) | 10 (1.8) | 5 (2.8) | 5 (1.4) | 0.307 |
| Outcomes up to week 48 | | | | |
| Death, n (%) | 0 (0) | 0 (0) | 0 (0) | - |
| Major relapse, n (%) | 0 (0) | 0 (0) | 0 (0) | - |
| Minor relapse, n (%) | 31 (5.7) | 7 (4.0) | 24 (6.5) | 0.244 |
| Severe infection, n (%) | 41 (7.5) | 20 (11.3) | 21 (5.7) | 0.025^*^ |

Data are presented as median [IQR] or as n (%), unless otherwise indicated.

AZA, Azathioprine; GC, Glucocorticoid; GFCR, Glucocorticoid-Free Clinical Remission; IVCYC, Intravenous Cyclophosphamide; MMF, Mycophenolate Mofetil; MTX, Methotrexate; MZR, Mizoribine; PLEX, Plasma Exchange; RTX, Rituximab.

For statistical analyses, **p* < 0.05, ***p* < 0.01. *P*-value: Wilcoxon rank sum test, Fisher’s exact test
